# Tanshinlactone triggers methuosis in breast cancer cells via NRF2 activation

**DOI:** 10.3389/fphar.2024.1534217

**Published:** 2025-01-21

**Authors:** Wanjun Lin, Zifeng Huang, Xuening Zhang, Dayuan Zheng, Yanchao Yang, Meina Shi, Dongfang Yang, Tong Chu, Wenzhe Ma

**Affiliations:** State Key Laboratory of Quality Research in Chinese Medicine, Macau University of Science and Technology, Macau, China

**Keywords:** tanshinlactone, breast cancer, catastrophic macropinocytosis, methuosis, NRF2 activation, drug resistance

## Abstract

**Background:**

Tanshinlactone is a compound derived from the herb *Salvia miltiorrhiza*. Breast cancer is the most prevalent malignancy among women globally. While significant strides have been made in breast cancer management, these interventions are often impeded by substantial adverse effects that undermine patients’ quality of life and confront limitations due to the eventual development of multi-drug resistance. Catastrophic macropinocytosis, also called methuosis, as a nonapoptotic cell death associated with cytoplasmic vacuolization, has gained increasing attention, largely because of its potential importance in cancer therapy.

**Methods:**

The effect of tanshinlactone on the growth of human cancer cells was evaluated using sulforhodamine B and colony formation assay. Fluorescent dyes are used to label macropinosomes and lysosomes. Phase contrast, confocal and transmission electron microscopy were employed to observe cell morphological changes. RT-PCR, western blot, lentiviral-mediated gene overexpression, and pharmacological inhibitor assays were comprehensively designed to regulate the identified signaling pathways and confirm the mechanism of tanshinlactone. Human breast cancer cell lines-derived xenograft tumor explants assay was used to evaluate the compound’s efficacy and to assess the induction of methuosis via NRF2 activation by tanshinlactone.

**Results:**

Tanshinlactone selectively inhibits the growth of ER+ and HER2+/EGFR + breast cancer cells while showing limited cytotoxicity against other cancer types and normal cells. The selective anti-breast cancer activity is associated with the induction of methuosis, characterized by cytoplasmic vacuolization due to dysfunctional macropinocytosis. This process is mediated by the activation of the transcription factor NRF2, leading to the formation of macropinosomes that fail to fuse with lysosomes or recycle to the plasma membrane, resulting in cell death. The *in vitro* induction of methuosis via NRF2 activation was replicated in a murine xenograft explants model. Additionally, tanshinlactone demonstrated effectiveness against lapatinib-resistant breast cancer cells, suggesting its potential as a therapeutic agent for overcoming drug resistance in cancer treatment.

**Conclusion:**

Tanshinlactone as a novel therapeutic agent, is capable of selectively inhibiting ER+ and HER2+/EGFR + breast tumors through a unique mechanism of inducing catastrophic macropinocytosis. This regimen holds promise for targeted therapy with minimized side effects and offers a new therapeutic avenue for breast patients with drug-resistant diseases.

## 1 Introduction

Breast cancer, the most prevalent malignancy worldwide, claims countless lives annually. Based on the expression levels of estrogen receptor (ER), progesterone receptor (PR), and human epidermal growth factor receptor 2 (HER2) (Brenton et al., 2005), breast cancer is classified into three molecular subgroups, ER+, HER2+, and triple-negative breast cancer (TNBC). Hormonal therapy, anti-HER2 agents, or chemotherapy constitute the current mainstay of systemic medical treatment for patients with breast carcinoma (Waks and Winer, 2019). Despite recent advances in prevention and treatment, breast cancer remains a formidable adversary, causing 670,000 deaths and 2.3 million new cases globally in 2022 (Bray et al., 2024). However, alongside these challenges, side effects from treatments and drug resistance persist, significantly impacting clinical outcomes (Shapiro and Recht, 2001; Tang et al., 2016). Consequently, there is an urgent need for novel anti-breast cancer agents with distinct mechanisms of action.

Macropinocytosis is an actin-driven, cholesterol-dependent form of endocytosis. The rearrangement of the actin cytoskeleton plays a crucial role in the formation of macropinosomes from ruffles that fold back on the plasma membrane (Nakase et al., 2004). Activated Rac1 localizes to the plasma membrane, where it stimulates the reorganization of actin filaments and membrane ruffling (Hall, 1998; Stow et al., 2020). The involvement of cholesterol is pivotal, as it facilitates the proper localization of active Rac1 to the membrane and supports actin-driven membrane activities, which are essential for macropinocytosis to occur (Grimmer et al., 2002; Ramirez et al., 2019). As a specialized mode of endocytosis, macropinocytosis orchestrates the engulfment of substantial extracellular fluid and particles by cells, encapsulating them within macropinosomes-vesicles ranging from 0.2 to 5 μm in size (Swanson, 2008). This process facilitates the internalization of diverse substances, including nutrients, growth factors, antigens, and therapeutic drugs (Lim and Gleeson, 2011). Remarkably, certain cancer cells, driven by oncogenic mutations, exploit macropinocytosis to secure nutrients, ensuring their survival and uncontrolled proliferation (Song et al., 2020). Consequently, inhibiting macropinocytosis has emerged as a promising therapeutic strategy to starve cancer cells and impede their growth (Masereel et al., 2003). Conversely, an intensified state of macropinocytosis, termed “methuosis”, leads to the formation of cytoplasmic fluid-filled vacuoles originating from macropinosomes. These vacuoles coalesce and rupture, ultimately causing non-apoptotic cell death in cancer cells, offering a potential avenue for treatment, especially in cases resistant to traditional chemotherapies (Maltese and Overmeyer, 2014).

The transcription factor nuclear factor erythroid 2-related factor 2 (NRF2) plays a dual role in cancer (Rojo de la Vega et al., 2018). As the master regulator of cellular antioxidant responses, transient NRF2 activation reduces susceptibility to chemical carcinogenesis (Kensler et al., 2007; Yang et al., 2016). Consequently, NRF2 agonists like bardoxolone, sulforaphane, and dimethyl fumarate have been explored for cancer and metabolic diseases (Dodson et al., 2019). However, NRF2 also activates oncogenes, leading to chemoresistance (Singh et al., 2006; Wang et al., 2008). Recent studies have unveiled another facet: cancer cells can bypass autophagy inhibition through NRF2-induced macropinocytosis, acquiring nutrients from the extracellular environment to fuel energy production and amino acid synthesis (Su et al., 2021a). This solidifies NRF2’s position as a paramount transcriptional activator for macropinocytotic machinery.

Tanshinlactone (TSL) was initially extracted from the herb *Salvia miltiorrhiza* (Luo et al., 1986), which was widely used in traditional Chinese medicine (TCM) for cardiovascular and cerebrovascular diseases, pain, and insomnia (Ryu et al., 1997; Wu et al., 1991). While its anti-cancer potential was first observed in HepG2 liver cancer cells by inhibiting tumor angiogenesis (Xiang et al., 2012), the full breadth of its anti-proliferative activities and the underlying mechanisms remain largely unexplored. Our investigation has illuminated a new facet of TSL’s pharmacological profile, revealing its selective anti-proliferative efficacy against breast cancer cells overexpressing estrogen receptor (ER) or human epidermal growth factor receptor 2 (HER2)/epidermal growth factor receptor (EGFR). Remarkably, TSL exhibits minimal toxicity towards normal cells and other types of cancer cells, emphasizing its favorable safety profile. This selective cytotoxicity is linked to the induction of methuosis via NRF2 activation. Furthermore, TSL exhibits efficacy against lapatinib-resistant breast cancer cells, offering a promising avenue for breast cancer therapy.

## 2 Materials and methods

### 2.1 Cell lines and culture conditions

Human breast cancer cell lines SK-BR-3, MDA-MB-453, BT-474, ZR-75-1, MCF7, T-47D, MDA-MB-468, MDA-MB-231, BT-549, HCC1937, human prostatic stromal myofibroblast cells WPMY-1, human glioblastoma cell lines U-87MG, human renal carcinoma cell lines 786-O, human fibrosarcoma cell lines HT-1080, murine breast cancer cell lines 4T1 were purchased from Cell Bank of the Chinese Academy of Sciences, Shanghai, China. Human normal breast epithelial cells MCF10A and human colon epithelial cells CCD841CoN were kind gifts from Prof. Lin Li at the Institute of Biochemistry and Cell Biology, Chinese Academy of Sciences, Shanghai, China. Human umbilical vein endothelial cells HUVECs, human colorectal cancer cell lines HCT116, LoVo, HCT-8, human lung cancer cell lines A549, HCC827, human rhabdomyosarcoma cell lines RD, A204, murine melanoma cell lines B16-F0 and human embryonic kidney cell line HEK293T were purchased from American Type Culture Collection (ATCC). The mouse embryonic fibroblast MEF from 13.5 to 14.5 ED pregnant c57BL/6 mice was isolated in our lab following the protocol from Perry J. Blackshear Lab (Qiu et al., 2016). The lapatinib-resistant breast cancer cell line SK-BR-3_LR600 was established in our lab by continuously exposed to lapatinib, starting with 10 nM and incrementally increasing to 600 nM over 20 months (Ma et al., 2010). To maintain the resistant phenotype, SK-BR-3_LR600 cells were routinely maintained in complete RPMI 1640 medium containing 600 nM of lapatinib. When for experimental purposes, to accurately assess the effects of treatments or to avoid any confounding influence of continuous lapatinib exposure, the growth medium is switched to complete RPMI 1640 devoid of lapatinib at least 48 h before conducting the experiments. The SK-BR-3, MDA-MB-453, BT-474, ZR-75–1, MCF7, T-47D, MDA-MB-468, MDA-MB-231, BT-549, HCC 1937, CCD841CoN, HCT-8, A549, HCC827, 786-O, 4T1 cells were cultured in RPMI 1640 medium (Gibco) supplemented with 10% fetal bovine serum (FBS, Gibco) and 1% Pen Strep Glutamine (100×, 10,000 Units/mL penicillin, 10,000 μg/mL streptomycin and 29.2 mg/ml L-glutamine) (Gibco). The WPMY-1, HUVECs, HCT116, LoVo, RD, A204, B16-F0, MEF, and HEK293T cells were cultured in Dulbecco’s modified Eagle medium (DMEM, Gibco) supplemented with 10% fetal bovine serum and 1% Pen Strep Glutamine. The U-87MG, HT-1080 cells were cultured in Minimum essential medium (MEM, Gibco) supplemented with 10% fetal bovine serum and 1% Pen Strep Glutamine. The MCF10A cells were cultured in specific epithelial culture medium (CM-0525, Procell Co., Ltd.). All cells were cultured in a humidified incubator with 5% CO_2_ at 37°C and routinely tested for *mycoplasma* by a PCR-based method (Young et al., 2010).

### 2.2 Reagents

Tanshilactone (TSL, YMbio, Shanghai, China), ethylisopropylamiloride (EIPA, MedChem Express), bafilomycin A1 (BafA1, MedChemExpress), methyl-β-cyclodextrin (mβCD, MedChemExpress), cytochalasin D (CytD, MedChemExpress), EHT1864 (MedChemExpress), ML385 (MedChemExpress), Q-VD-OPh (QVD, MedChemExpress), liproxstatin-1 (Lip-1, MedChemExpress), necrostatin-1 (Nec-1, MedChemExpress) and lapatinib (L, MedChemExpress) were dissolved in dimethyl sulfoxide (DMSO, Sigma Aldrich) and stored at −40°C.

### 2.3 Antibodies

Primary antibodies sources were as follows: Rabbit monoclonal to NRF2 (#ab62352), rabbit polyclonal to NQO1 (#ab34173) anti-bodies were purchased from Abcam. Rabbit monoclonal to HO-1 (#5853) anti-body was purchased from Cell Signaling Technology. Mouse monoclonal anti-β-actin (#A5441) anti-body was purchased from Sigma Alarich. Mouse monoclonal to Ki67 (#sc-23900) anti-body was purchased from Santa Cruz Biotechnology. Anti-mouse (#715-035-150) and anti-rabbit (#211-035-109) secondary antibodies were purchased from Jackson ImmunoResearch Laboratories.

### 2.4 Cell proliferation assay

Cell proliferation assays were performed using Sulforhodamine B (SRB) colorimetric assay, as previously described (Lin et al., 2016). Cells were seeded in 96-well plates in a volume of 100 μL/well at densities of 0.5–1 × 10^4^ cells per well and incubated overnight. Then 100 μL of medium containing the indicated drugs (2 × indicated concentrations) was added. After incubation for 72 h, the attached cells were fixed with 50 μL of cold 50% (w/v) trichloroacetic acid (TCA, Sigma Aldrich) at 4°C for 1 h and subsequently stained with 100 μL of 0.4% (w/v) SRB (Sigma Aldrich) at room temperature for 30 min. The OD determination at 515 nm was determined using a SpectraMax 190 microplate reader (Molecular Devices) following the addition of 200 μL of 10 mM Tris base solution (pH 10.5, Sigma Aldrich).

### 2.5 Colony formation assay

Colony formation assay was carried out as previously described (Lin et al., 2017). Briefly, cells were plated at densities of 1–2.5 × 10^3^ cells/well or 1–2 × 10^5^ cells/well in 6-well plates and incubated in different doses of the indicated drugs for 7–14 days or incubated in drug-containing medium for 48 h, then continuously cultured in drug-free medium for 7–14 days. Surviving colonies were washed with phosphate buffer saline (PBS, Invitrogen), stained with 0.2% (w/v) crystal violet (Sigma Aldrich) in buffered formalin (Sigma Aldrich) for 10 min, and photographed using a GelDoc XR Imaging System (Bio-Rad). The colony numbers (diameter >50 μm) were counted using ImageJ software.

### 2.6 Flow cytometric analysis of cell cycle

Cells were seeded in 6-well plates at a density of 1–2 × 10^5^ cells/mL and treated with the indicated drugs for 48 h. Cells were harvested, washed twice with PBS, fixed using 70% ethanol at −20°C for 2 h, and stained for total DNA content with a PI solution containing 20 μg/mL propidium iodide (PI), 200 μg/mL DNase-free RNase A, and 0.1% triton X-100 in PBS for 30 min at room temperature. Cell cycle distribution was analyzed by FACSAria II flow cytometry (BD Bioscience) and the percentage of the total cell population in the three different phases (G0/G1, S, G2/M) of cell cycle was determined using FlowJo software.

### 2.7 Flow cytometric analysis of apoptosis

Cellular apoptosis was analyzed with a FITC Annexin V Apoptosis Detection Kit I (BD Bioscience) by FACSAria II flow cytometry. Briefly, cells were plated in 6-well plates at a density of 1–2 × 10^5^ cells/mL and treated with the indicated drugs. After 48 h of treatment, cells were harvested, washed twice with cold PBS, and resuspended in 100 μL of 1 × binding buffer, stained with 5 μL of FITC Annexin V and 5 μL PI, and incubated for 15 min in the dark at room temperature. Cells were filtered and analyzed by flow cytometry within 1 h. Total apoptotic cells were counted using FlowJo software.

### 2.8 Assessment of cell morphological changes

Cells were grown in 96-well culture plates at densities of 0.5–1 × 10^4^ cells per well and treated with test compounds or vehicles for different doses or durations. Cell phenotypic alteration (cell vacuolization) was visualized through a phase contrast microscope (Olympus CKX53) at a 20 × objective.

### 2.9 Time-lapse microscopy

Cells were cultured on 35 mm glass-bottom dishes (SPL) and treated with test compounds. Then the dishes were placed in a humidified live cell chamber equilibrated with 5% CO_2_ at 37°C. Phase-contrast images at a 40 × objective were acquired automatically for the indicated period of time by API Delta Vision Live-Cell Imaging System.

### 2.10 Transmission electron microscopy (TEM)

Transmission Electron Microscopy assay was carried out as described previously (Winey et al., 2014). In brief, cells were plated in 100 mm dishes at a density of 1–2 × 10^6^ cells/mL and treated with the indicated drugs or vehicle. Cell pellets were fixed in 2.5% glutaraldehyde (Electron Microscope Sciences) at 4°C overnight and post-fixed for 2 h with 1% OsO4 (Electron Microscope Sciences) after washed with PBS for 6 h. Dehydration was carried out by a graded series of ethanol solutions (30%–100%, Anaqua), followed by a final dehydration with propylene oxide (Sigma). Then the pellets were infiltrated and embedded in epoxy resin (Electron Microscope Sciences). Ultrathin sections were obtained and collected on copper support grids, followed by uranyl acetate (Electron Microscope Sciences) and lead citrate trihydrate (Electron Microscope Sciences) staining. Images were captured with a JEOL JEM-1400 series 120 kV Transmission Electron Microscope.

### 2.11 Confocal microscopy

Confocal images were captured using a Zeiss LSM SP8 laser confocal microscope (Zeiss LSM). A total of 1–2 × 10^5^ cells were cultured on 35 mm confocal dish (glass-bottom dishes, SPL), treated with DMSO or test compounds for indicated times, and imaged with a 40/63 × oil objective.

Labeling of macropinosomes with fluid-phase fluorescent tracer FITC-dextran (70 kDa) followed the protocol as described previously (Commisso et al., 2014). Untreated and TSL-treated cells were incubated with 1 mg/mL of FITC-dextran (70 kDa) (Invitrogen) in the appropriate serum-free medium in a 37°C, 5% CO_2_ incubator for 30 min. Then the dextran cell culture incubation media was carefully aspirated, and the cells were washed two times with ice-cold PBS. Bright light and fluorescent images were captured using confocal microscope. To visualize the lysosomes, the cells were subsequently incubated for 15 min in the fresh medium with 75 nM of LysoTracker Red DND-99 (Invitrogen), washed twice with fresh medium, and then imaged with FITC and TRITC filters.

Labeling of intracellular vacuoles with fluid-phase fluorescent tracer lucifer yellow was performed as described (Srivastava et al., 2019). Untreated and TSL-treated cells were incubated with 1.25 mg/mL of lucifer yellow (LY, Sigma) in Hanks balanced salt solution (HBSS, Gibco) in a 37°C, 5% CO_2_ incubator. After 30 min, the lucifer yellow was removed and the cells were washed two times with HBSS buffer. Bright light and fluorescent images were captured using confocal microscope.

### 2.12 Flow cytometric analysis of FITC-dextran (70 kDa) uptake

Non-microscopy-based quantitative method for the analysis of macropinocytosis utilizes Flow Activated Cell Sorting (FACS). Briefly, cells were seeded at 2–4 × 10^5^ cells/well in 6-well-plate overnight. Untreated and TSL-treated cells were incubated with 1 mg/mL of FITC-dextran (70 kDa) in the appropriate serum-free medium in a 37°C, 5% CO_2_ incubator for 30 min. Then cells are washed, trypsinized, and dextran uptake is subsequently quantified via a FACSAria II flow cytometry (BD Bioscience). The data were analyzed using FlowJo software to determine the total number of dextran-positive cells. Mean fluorescence intensity of the population was determined.

### 2.13 Flow cytometric analysis of fluid-phase fluorescent tracer lucifer yellow uptake

The uptake of lucifer yellow was quantified by flow cytometry (Overmeyer et al., 2008). Briefly, cells were seeded at 2–4 × 10^5^ cells/well in 6-well-plate overnight. Untreated and TSL-treated cells were incubated with 1.25 mg/mL of lucifer yellow in HBSS in a 37°C, 5% CO_2_ incubator for 30 min. Uptake was stopped by washing the cells two times with HBSS. Then cells were harvested by trypsinization, washed with HBSS, and resuspended in 500 ul of HBSS. For each sample, 10,000 events were analyzed with a Beckman-Coulter CytoFLEX flow cytometer. The data were analyzed using FlowJo software to determine the total number of lucifer yellow-positive cells. The mean fluorescence intensity of the population was determined.

### 2.14 Lentiviral transduction and stable cell line generation

pLV2-CMV-Rab5A-mCherry, pLV3-CMV-Rab7-mCherry, and pLV3-CMV-Rab11A-EGFP lentiviral constructs were used to express fusion protein Rab5A-mCherry, Rab7-mCherry, and Rab11A-EGFP in target cells respectively. To generate lentivirus, HEK293T cells were seeded at 1 × 10^6^ cells/well in 6-well plates and incubated overnight before being transfected with pLV2-CMV-Rab5A-mCherry/pLV3-CMV-Rab7-mCherry/pLV3-CMV-Rab11A-EGFP (0.5 μg), psPAX2 (0.375 μg) and pMG2. G (0.125 μg) in 50 μL of Opti-MEM (Gibco) with 3 μL of FuGENE HD (Promega). The transfection mixture was incubated for 5–10 min at room temperature and then added dropwise to the HEK293T cells. After 16 h, the medium containing the transfection mixture was replaced with fresh full medium. Supernatants were collected, filtered through 0.45 μm filters (Sigma), and directly added to target cells or stored at −80°C for later use after an additional 24 h. Lentiviral transduction was performed in the presence of 5–10 μg/mL of polybrene (Sigma). After 12–16 h incubation, the viral transduction medium was exchanged with fresh medium with antibiotic selection markers to generate positive cells.

### 2.15 Quantitative real-time PCR

Cells were cultured on 24-well-plate and treated with different doses of compound for 24 h. Cellular mRNA was purified by binding to poly (dT) magnetic beads (Dynal) and reverse transcribed using SuperScript III (Invitrogen) by the manufacturer. Quantitative real-time PCR was performed in duplicates three times by using SYBR Green (Molecular Probes) on the ViiA™ 7 Real-Time PCR System (Applied Biosystems). Relative mRNA levels were quantified and normalized to the eukaryotic translation initiation factor (TIF) expression level by subtracting the cycling threshold for the control from the threshold for the target. The primer sequences can be obtained upon request.

### 2.16 Western blotting

Cells were plated in 6-well plates and treated with the indicated drugs for 48 h. Protein samples were lysed in RIPA buffer with protease inhibitor cocktail (Roche) and phosphatase inhibitor (Roche). Samples were boiled at 95°C for 5 min after dilution in SDS-PAGE protein sample buffer. Each protein sample (20 μg) was loaded on sodium dodecyl sulfate-polyacrylamide (SDS-PAGE) gels. The gels were run for 30 min at 80 V for stacking and then added to 125 V for protein separation. After fractionation on SDS-PAGE gels, the proteins were transferred to polyvinylidene difluoride (PVDF) membranes (Millipore) for 2 h at 300 mA. The membranes were blocked with 5% bovine serum albumin (BSA, Roche) diluted with 1 × Tris buffered saline with 0.1% Tween 20 (TBST) at room temperature for 1 h and incubated with primary antibodies overnight at 4°C. Then the membranes were washed three times with 1 × TBST and incubated with the appropriate horseradish peroxidase (HRP)-conjugated secondary antibody at room temperature for 45 min. Proteins were visualized with SuperSignal West Dura Extended Duration Substrate, SuperSignal West Pico Chemiluminescent Substrate or SuperSignal West Femto Tial Kit (Thermo Scientific). β-actin was used as endogenous loading control for normalization.

### 2.17 Human breast cancer cell lines derived xenograft tumor explants assay

Female BALB/c athymic nude mice (4–6 weeks old) were subcutaneously injected with 2 × 10^6^ of ZR-75–1 cells suspended in 100 μL of PBS into hind limb. When reaching to suitable size (300 ∼ 500 mm^3^), the tumor specimens were removed, transported to cold PBS on ice, sliced into approximately 2–3 mm^3^ sections, and placed into the wells of 24-well-plate containing 500 μL of complete RPMI 1640 medium with DMSO or TSL under sterile conditions and typically within 1–2 h of surgery. The plate was cultured at 37°C and 5% CO_2_ for various time points or concentrations, after which, histocultures were harvested for the desired downstream analysis (Centenera et al., 2022; Holliday et al., 2013).

For HE staining, tissue was sectioned at 3 μm thickness, then stained with hematoxylin and eosin following the manufacturer of HE staining kit (abcam). Slides were visualized using a microscope equipped with a Leica DFC310 FX digital color camera at a 20 × objective.

For IHC staining, tissue sections underwent heat-induced epitope retrieval in sodium citrate buffer (10 mM, pH 6.0, Sigma) for 20 min at 95°C. Then sections were blocked in 3% BSA in PBS for 30 min and probed with primary antibody (1:100) for 1 h. Endogenous peroxidase activity was quenched, and detection was performed using the HRP-conjugated secondary antibody followed by colorimetric detection using diaminobenzidine (Thermo). Tissues were counterstained with hematoxylin. Images were captured on a microscope equipped with a Leica DFC310 FX digital color camera at a 20 × objective.

For FITC-dextran (70 kDa) staining, tissues were prepared using frozen techniques (Commisso et al., 2014). DMSO and TSL-treated tumor sections were incubated with 1 mg/mL of FITC-dextran in the appropriate serum-free medium in a 37°C, 5% CO_2_ incubator for 1 h. Then the dextran culture incubation media was carefully aspirated, and the sections were washed two times with ice-cold PBS, covered completely with Tissue-Tek O.C.T compound (Sakura) in a cryomold, and placed atop dry ice for 10–15 min, stored at −80°C for at least 24 h. The frozen tissue sections were serially sliced at 6–8 μm thickness, rinsed in PBS immediately prior to fixation in formaldehyde solution (Sigma), and subjected to DAPI staining after three PBS washes for 3 min. All operations were performed avoiding light. Images were visualized through DAPI and FITC filters of fluorescence inverted microscope (Leica DM2500 Fluorescence Microscope) at 20 × objective.

For WB analysis, tumor sections were homogenized in RIPA buffer with protease inhibitor cocktail and phosphatase inhibitor on ice, constantly agitated for 2 h at 4°C, centrifuged for 20 min at 15,000 g at 4°C, and the supernatant was aspirated for protein analysis by SDS-PAGE Gel Electrophoresis.

For RT-PCR analysis, frozen tumor sections were ground in liquid nitrogen quickly, lysed and homogenized rapidly in lysis/binding buffer (Invitrogen). mRNA purification and RT-PCR analysis were proceeded as previously described (Lin et al., 2017).

### 2.18 In silico ADME properties prediction

The QikProp software, developed by Professor Bill Jorgensen’s lab at Yale University (Jorgensen and Duffy, 2002), was used to predict the ADME properties of TSL within the Schrödinger Maestro. Additionally, the molecular properties of TSL were compared with those of 95% of known drugs using the same QikProp tool.

### 2.19 Statistical analysis

Data are shown as mean ± SD and the statistical significance was determined by two-tailed Student’s t-test. The data were analyzed by one-way analysis of variance (ANOVA) when more than two groups were compared (*, represents P < 0.05, **, represents P < 0.01, ***, represents P < 0.001).

## 3 Results

### 3.1 Selective inhibition of breast cancer cell proliferation by tanshinlactone

**FIGURE 1:**
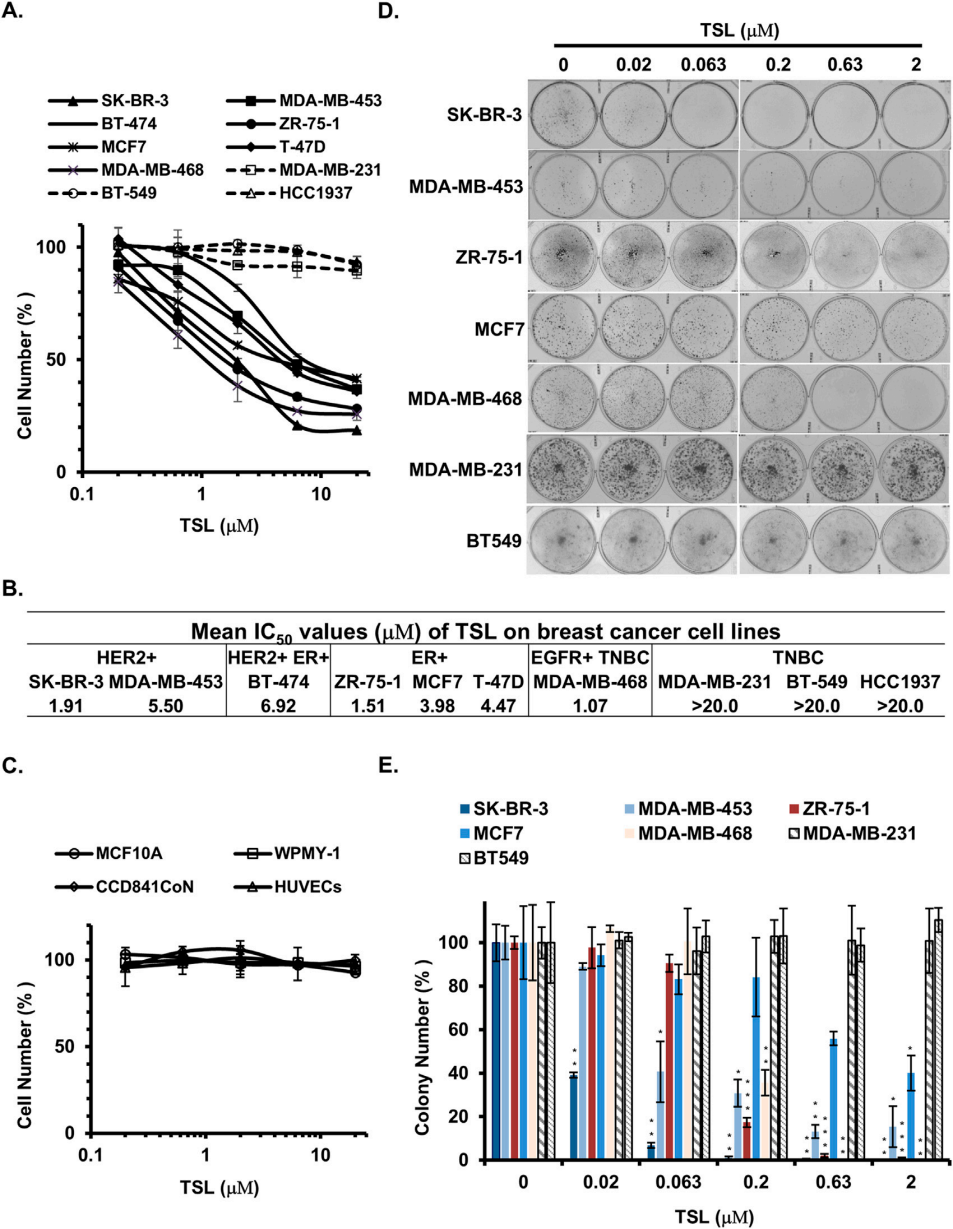
Tanshinlactone selectively inhibits the proliferation of ER+ and HER2+/EGFR + breast cancer cells. Human breast cancer cell lines, human normal breast epithelial cells, human prostatic stromal myofibroblast cells, human colon epithelial cells, human umbilical vein endothelial cells, and human hepatocyte cells were used to determine the growth inhibition effect of TSL. **(A)** Dose effect of TSL treatment (72 h) on the proliferation of human breast cancer cell lines (SK-BR-3, MDA-MB-453, BT-474, ZR-75-1, MCF7, T-47D, MDA-MB-468, MDA-MB-231, BT-549 and HCC1937). **(B)** Mean IC_50_ values of TSL on breast cancer cell lines. **(C)** Dose effect of TSL treatment (72 h) on the proliferation of human normal breast epithelial cells (MCF10A), human prostatic stromal myofibroblast cells (WPMY-1), human colon epithelial cells (CCD841CoN), human umbilical vein endothelial cells (HUVECs). The cell number at each TSL concentration is represented as a percentage of control (no TSL treatment). Average values are from three independent experiments performed in duplicate (n = 3). **(D)** Colony formation of breast cancer cell lines SK-BR-3, MDA-MB-453, ZR-75-1, MCF7, MDA-MB-468, MDA-MB-231, and BT549 after treatment with different doses of TSL for 7–14 days. Representative colony formation assay plates are shown (n = 3). **(E)** Colony formation plates were quantified by counting colony number (n = 3). Data are shown as mean ± SD. P-values determined by Student’s t-test compared to control. *P < 0.05; **P < 0.01; ***P < 0.001.

To conduct a comprehensive assessment of the *in vitro* anti-proliferative properties of tanshinlactone (TSL), we utilized a varied selection of breast cancer cell lines in the Sulforhodamine B (SRB) assay (Table 1). This was done in conjunction with normal cell lines and other cancer cell lines derived from both human and mouse sources (Table 2). Our results showed that TSL inhibited the growth of HER2+ and ER + breast cancer cell lines in a dose-dependent manner (Figure 1A). However, TSL did not affect triple-negative breast cancer (TNBC) cell lines (Figure 1A), normal cell lines (Figure 1C, Supplementary Figure S1I), or other types of cancer cell lines (Supplementary Figures S1A–H). Interestingly, MDA-MB-468, a TNBC cell line, was highly sensitive to TSL treatment (Figure 1A). We analyzed the gene expression profile obtained from the Cancer Cell Line Encyclopedia (CCLE) database and confirmed that epidermal growth factor receptor 1 (HER1 or EGFR), which contributes to the development and progression of breast cancer similar to HER2 (Nuciforo et al., 2015; Witton et al., 2003), is highly expressed in MDA-MB-468 cells (Supplementary Figure S1J).

**TABLE 1:** Molecular subtypes of breast cancer cell lines used in our study.

Breast cancer cell line classification										
Cell line	Oncotree subtype and code	Type	ER	PR	HER2	Other types of ERBB	Ras mutation	NRF2 mutation	Other types of mutation	Therapy
SK-BR-3	Invasive Breast Carcinoma (BRCA)	HER2+ (15%–20%)	-	-	+	-	-	-	TP53	Target therapy
MDA-MB-453	Invasive Breast Carcinoma (BRCA)	HER2+ (15%–20%)	-	-	+	-	-	-	TP53, PIK3CA, PTEN	Target therapy
BT-474	Invasive Ductal Carcinoma (IDC)	Luminal HER2 (5%–10%)	+	+	+	-	-	-	TP53, PIK3CA, BRCA2	Endocrine therapy; Target therapy
ZR-75–1	Invasive Ductal Carcinoma (IDC)	Luminal A (40%)	+	+	-	-	HRAS (E162K)	-	PTEN	Endocrine therapy
MCF7	Invasive Breast Carcinoma (BRCA)	Luminal A (40%)	+	+	-	-	-	-	PIK3CA	Endocrine therapy
T-47D	Invasive Ductal Carcinoma (IDC)	Luminal A (40%)	+	+	-	-	-	-	TP53, PIK3CA	Endocrine therapy
MDA-MB-468	Invasive Breast Carcinoma (BRCA)	Basal-like (∼10%)	-	-	-	EGFR+	-	-	TP53, BRCA2	Chemotherapy
MDA-MB-231	Invasive Breast Carcinoma (BRCA)	Normal-like (∼5%)	-	-	-	-	KRAS (G13D)	-	TP53	Chemotherapy
BT-549	Invasive Carcinoma, NOS (BRCNOS)	Normal-like (∼5%)	-	-	-	-	-	-	TP53, PTEN	Chemotherapy
HCC1937	Invasive Ductal Carcinoma (IDC)	Basal-like (∼10%)	-	-	-	-	-	-	TP53, BRCA1	Chemotherapy

**TABLE 2:** Classification of cell lines used in our study.

Cell line classification							
Cell line	Cell type	Category	Oncotree primary disease	Organism	Tissue	Morphology	Mutation
MCF10A	Human normal breast epithelial cells	Non-tumorigenic cell line		*Homo sapiens*	Breast; Mammary gland	Ehpithelial cell	
WPMY-1	Human prostatic stromal myofibroblast cells	Non-tumorigenic cell line	-	*Homo sapiens*	Prostate; Stroma	Epithelial, fibroblast, myofibroblast cell	
CCD841CoN	Human colon epithelial cells	Non-tumorigenic cell line	-	*Homo sapiens*	Large intestine; Colon	Ehpithelial cell	
HUVECs	Human umbilical vein endothelial cells	Non-tumorigenic cell line	-	*Homo sapiens*	Umbilical cord; Umbilical vein; Vascular endothelium	Ehpithelial cell	
MEF	Mouse embryonic fibroblast	Non-tumorigenic cell line	-	*Mus musculus*	Embryo	fibroblast	
HCT116	Human colorectal cancer cell lines	Cancer cell line	Colorectal adenocarcinoma	*Homo sapiens*	Large intestine; Colon	Ehpithelial cell	KRAS (G13D), PIK3CA(H1047R)
LoVo	Human colorectal cancer cell lines	Cancer cell line	Colorectal adenocarcinoma	*Homo sapiens*	Large intestine; Colon	Ehpithelial cell	KRAS (G13D)
HCT-8	Human colorectal cancer cell lines	Cancer cell line	Colorectal adenocarcinoma	*Homo sapiens*	Large intestine; Colon	Ehpithelial cell	
A549	Human lung cancer cell lines	Cancer cell line	Non-Small cell lung cancer	*Homo sapiens*	Lung	Ehpithelial cell	KRAS (G12S), KEAP1 (G333C)
HCC827	Human lung cancer cell lines	Cancer cell line	Non-Small cell lung cancer	*Homo sapiens*	Lung	Ehpithelial cell	EGFR (E746_A750del), TP53(V218del)
U-87MG	Human glioblastoma cell lines	Cancer cell line	Diffuse glioma	*Homo sapiens*	Brain	Ehpithelial cell	
786-O	Human renal carcinoma cell lines	Cancer cell line	Renal cell carcinoma	*Homo sapiens*	kidney	Ehpithelial cell	TP53 (R248W), PTEN (Q149Ter)
HT-1080	Human fibrosarcoma cell lines	Cancer cell line	Fibrosarcoma	*Homo sapiens*	Connective tissue	Ehpithelial cell	NRAS (Q61K), IDH1 (R132C)
RD	Human rhabdomyosarcoma cell lines	Cancer cell line	Rhabdomyosarcoma	*Homo sapiens*	Muscle	Spindle cells and large multinucleated cells	NRAS (Q61H), TP53 (R248W)
A204	Human rhabdomyosarcoma cell lines	Cancer cell line	Rhabdoid cancer	*Homo sapiens*	Muscle	Ehpithelial cell	
4T1	Murine breast cancer cell lines	Cancer cell line	Mimics human triple-negative breast cancer	*Mus musculus*	Breast; Mammary gland	Ehpithelial cell	
B16-F0	Murine melanoma cell lines	Cancer cell line	Melanoma	*Mus musculus*	Skin	Mixture of spindle-shaped and epithelial-like cells	

Our findings suggest that TSL selectively inhibits the proliferation of breast cancer cells that highly express ER or HER2/EGFR, given the IC_50_ values of TSL presented in Figure 1B. To validate this selective activity, we performed a colony formation assay. SK-BR-3 (HER2+), MDA-MB-453 (HER2+), ZR-75–1 (ER+), MCF7 (ER+), and MDA-MB-468 (EGFR+) cells exhibited a substantial decline in colony formation capabilities following exposure to TSL. In contrast, the MDA-MB-231 and BT549 cells, characterized by the absence of these gene expressions, exhibited no significant alterations under analogous treatment conditions (Figures 1D, E).

### 3.2 Tanshinlactone induces extensive vacuolization in sensitive breast cancer cells

The induction of cell cycle arrest and apoptosis are the principal outcomes of cancer therapy, leading to diminished cell proliferation and subsequent cell death. So, we first analyzed the impact of TSL on cell cycle progression using TSL-sensitive breast cancer cell lines SK-BR-3, ZR-75–1, and MDA-MB-468, as well as TSL-insensitive cells MDA-MB-231 as models. As shown in Supplementary Figures S2A–C, the cell cycle distribution of SK-BR-3, ZR-75–1, and MDA-MB-468 cells remained largely unchanged after 48 h of TSL treatment at various concentrations (0, 2, 6.32, 20 μM). We then assessed whether apoptosis could explain TSL’s selective anti-proliferative activity on breast cancer cells. Flow cytometry revealed a slight increase in the number of apoptotic cells in sensitive cell lines (SK-BR-3, ZR-75-1, and MDA-MB-468) compared to the insensitive MDA-MB-231 cells following 48 h of TSL treatment (Supplementary Figures S3A–D). However, co-treatment with Q-VD-OPh (QVD), a broad-spectrum caspase inhibitor, did not alleviate TSL-induced cell death (Supplementary Figure S4A). This suggests that most of the cell death induced by TSL is caspase-independent, and the minor apoptotic response may be a compensatory stress reaction. Notably, neither liproxstatin-1 (Lip-1, a ferroptosis inhibitor) nor necrostatin-1 (Nec-1, a necroptosis inhibitor) could prevent cell death induced by TSL in SK-BR-3 and ZR-75-1 cells (Supplementary Figures S4B–C), ruling out these cell death modes.

**FIGURE 2:**
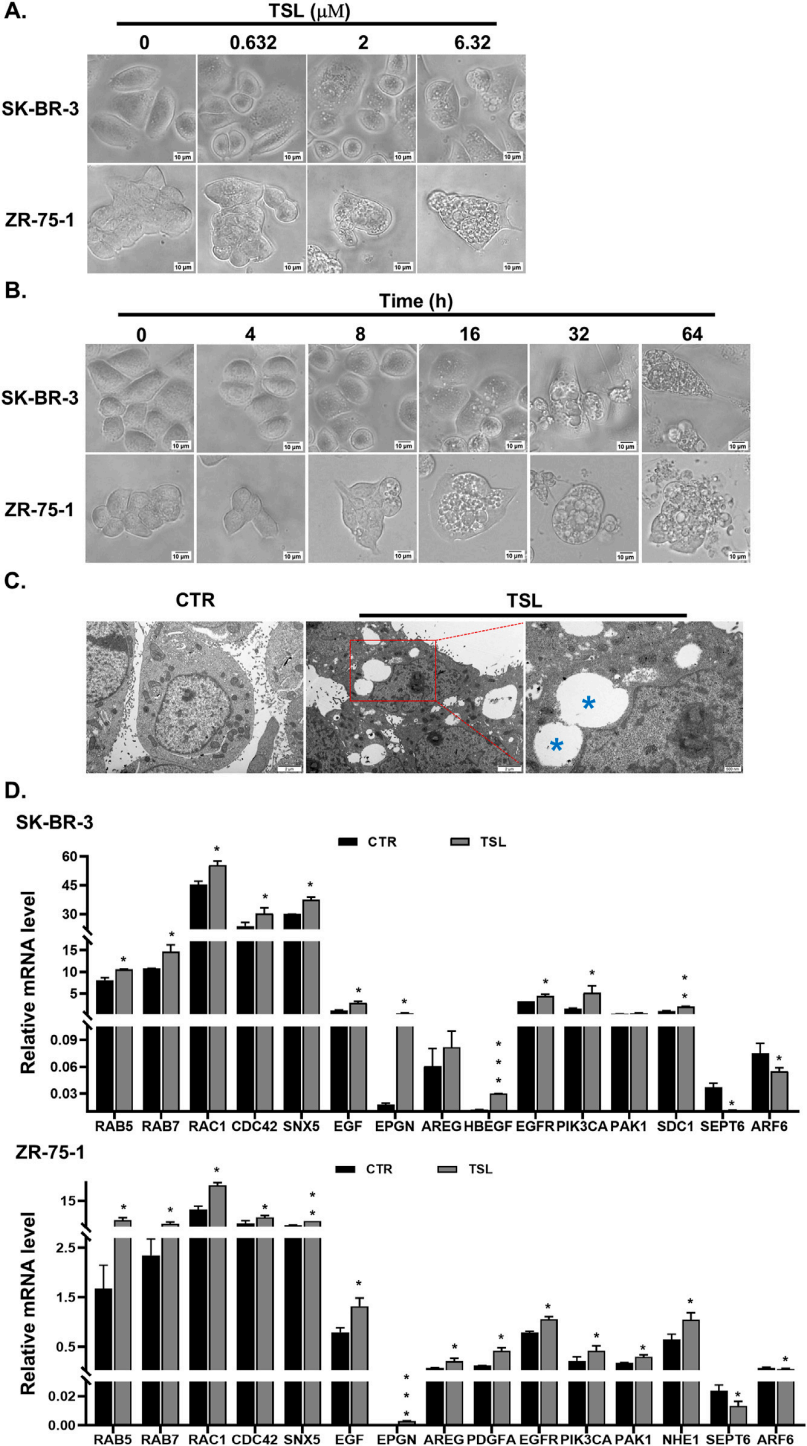
Tanshinlactone induces extensive vacuolization in breast cancer cell lines SK-BR-3 and ZR-75-1. **(A)** Phase-contrast images showing dose-dependent effects on cytoplasmic vacuolization of SK-BR-3 and ZR-75-1 cells following TSL (0, 0.632, 2 and 6.32 μM, 24 h) treatment (20×). Representative images are shown (n = 3). The scale bar is 10 μm. **(B)** Representative phase-contrast images showing time-dependent effects on vacuoles emergent, aggregation and cell rupture in TSL-treated SK-BR-3 and ZR-75-1 cells (6.32 μM, 0, 4, 8, 16, 32, 64 h) (20×) (n = 3). The scale bar is 10 μm. **(C)** Representative TEM images of DMSO-treated and TSL-treated (6.32 μM, 24 h) ZR-75-1 cells (n = 3). Control cells showing well-maintained cytoplasmic compartments. TSL-treated cells displaying massive cytoplasmic vacuolization varied in size. The scale bar is 2 μm. The enlarged image showing TSL-induced vacuoles appearing mostly empty bound by single membrane (blue asterisks). The scale bar is 500 nm. **(D)** Quantitative real-time PCR analyses of mRNA levels of *RAB5, RAB7, RAC1, CDC42, SNX5, EGF, EPGN, AREG, HBEGF, PDGFA, EGFR, PIK3CA, PAK1, NHE1, SDC1, SEPT6* and *ARF6* in SK-BR-3 and ZR-75-1 cells after treatment with TSL (6.32 μΜ) for 24 h. Average values are from three independent experiments performed in duplicate (n = 3). Data are shown as mean +SD. P-values determined by Student’s t-test compared to control. *P < 0.05; **P < 0.01; ***P < 0.001.

Interestingly, we observed substantial cytoplasmic vacuolization in SK-BR-3 and ZR-75-1 cells following TSL treatment (Figure 2A). Similar effects were seen in other responsive cell lines (MDA-MB-453, BT-474, MCF7, T-47D, and MDA-MB-468), but not in the insensitive MDA-MB-231 cells (Supplementary Figure S5A). Transmission electron microscopy (TEM) confirmed this vacuolization in TSL-treated ZR-75-1 cells compared to control (Figure 2C). Most of the large vacuoles were empty and enclosed by a single membrane (Figure 2C, denoted by blue asterisks). These vacuoles began to appear after 8 h of exposure to 6.32 μΜ of TSL, increasing in number and size over time, eventually merging to form massive vacuoles that led to cell death (Figure 2B and Supplementary Movie S1). These changes are similar to methuosis-like cell death associated with catastrophic macropinocytosis (Overmeyer et al., 2011; Sun et al., 2017). A gene signature has been identified in macropinocytosis (Su et al., 2021a), encompassing markers for endosomes, genes involved in the formation of filopodia and lamellipodia, as well as growth factors and receptors that stimulate this process. Specifically, tyrosine kinase receptors and their ligands, including EGFR and its ligands epidermal growth factor (EGF), epiregulin (EPGN), amphiregulin (AREG), and heparin-binding EGF-like growth factor (HBEGF) were significantly induced by TSL in SK-BR-3 and ZR-75-1 cells (Figure 2D). This is in line with their roles in actin-mediated membrane ruffling and macropinosome formation (Bryant et al., 2007; Henriksen et al., 2013; Lee et al., 2019; Lim and Gleeson, 2011; Nakase et al., 2015; Roepstorff et al., 2009; Wieduwilt and Moasser, 2008), and the selective activity of TSL on breast cancer cell lines (Figure 1A).

### 3.3 Tanshinlactone induces a dysfunctional macropinocytotic process in sensitive breast cancer cells

**FIGURE 3:**
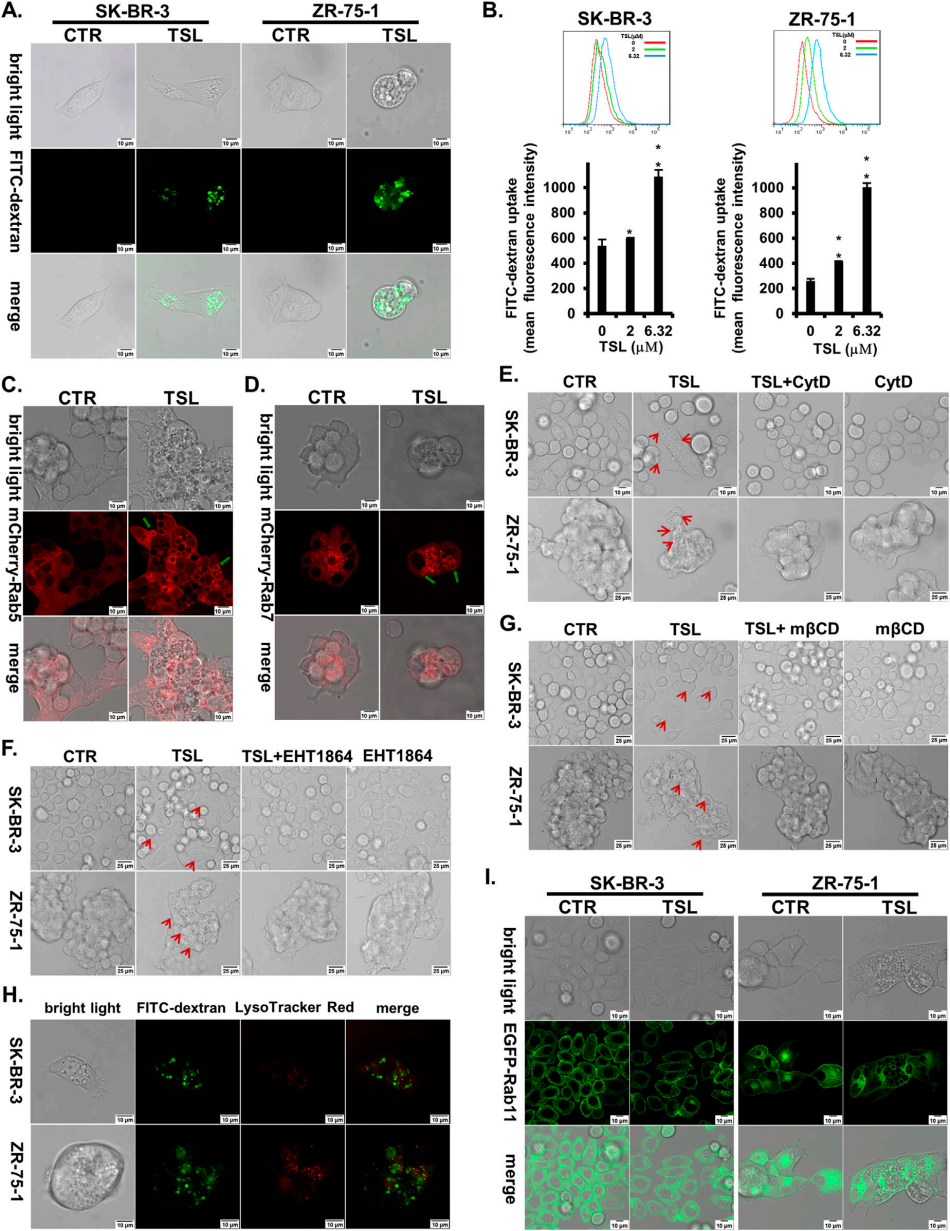
Tanshinlactone induces a dysfunctional macropinocytotic process in breast cancer cell lines SK-BR-3 and ZR-75-1. **(A)** Overlay of bright field microphotographs on FITC-dextran (70 kDa) accumulation in DMSO or TSL (6.32 μΜ) treated SK-BR-3 and ZR-75-1 cells (63×, zoom 2) at 24 h. The scale bar is 10 μm. **(B)** Quantitative FITC-dextran (70 kDa) uptake by SK-BR-3 and ZR-75–1 cells determined by flow cytometry after being treated with TSL (0, 2, 6.32 μΜ) for 24 h. Average values are from three independent experiments performed in duplicate (n = 3). Data are shown as mean ± SD. P-values determined by Student’s t-test compared to control. *P < 0.05; **P < 0.01; ***P < 0.001. **(C)** Confocal images showing the location of Rab5 on macropinosomes after treatment of TSL (6.32 μΜ, 24 h) in ZR-75-1 expressing mCherry-Rab5 (40×, zoom 3). The scale bar is 10 μm. Green arrows indicating Rab5+ macropinosomes. **(D)** Confocal images showing the location of Rab7 on macropinosomes after treatment of TSL (6.32 μΜ, 24 h) in ZR-75-1 expressing mCherry-Rab7 (40×, zoom 3). The scale bar is 10 μm. Green arrows indicating Rab7+ macropinosomes. **(E)** Confocal microphotographs showing that TSL (6.32 μΜ, 24 h) induced macropinosomes of SK-BR-3 and ZR-75-1 cells with or without Cytochalasin D treatment (0.1 μΜ, 24 h) (63×). The scale bar for SK-BR-3 cells is 10 μm, and for ZR-75–1 cells is 25 μm. Red arrows indicating the formation of vacuoles. **(F)** Confocal microphotographs showing that TSL (6.32 μΜ, 24 h) induced macropinosomes of SK-BR-3 and ZR-75–1 cells with or without EHT1864 treatment (25 μΜ,24 h) (63×). The scale bar is 25 μm. Red arrows indicating the formation of vacuoles. **(G)** Confocal microphotographs showing that TSL (6.32 μΜ, 24 h) induced macropinosomes of SK-BR-3 and ZR-75-1 cells with or without cholesterol inhibitor mβCD treatment (1 mM, 24 h) (63×). The scale bar is 25 μm. Red arrows indicating the formation of vacuoles. **(H)** Macropinocytotic cells SK-BR-3 and ZR-75-1 induced by TSL (6.32 μΜ, 24 h) were subjected to confocal microscopy to localize macropinosomes staining with FITC-dextran (70 kDa) and lysosomes staining with LysoTracker Red (63×, zoom 3). The scale bar is 10 μm. **(I)** Confocal images showing the location of Rab11 on macropinosomes after treatment of TSL (6.32 μΜ, 24 h) in SK-BR-3 and ZR-75-1 cells expressing Rab11-EGFP (40×, zoom 2). The scale bar is 10 μm. Representative images are shown (n = 3).

To confirm and quantify TSL-induced macropinocytosis, we used a FITC-labelled dextran (70 kDa) probe. When added to the medium, most vacuoles in TSL-treated cells were positive for dextran (Figure 3A). Flow cytometry further showed that TSL enhanced the internalization of the dextran in SK-BR-3 and ZR-75–1 cells in a dose-dependent manner (Figure 3B). This was replicated using another fluorescent dye, lucifer yellow (Supplementary Figures S6A–D). To further demonstrate the macropinosomes induced by TSL treatment, we transfected ZR-75-1 cells with mCherry-Rab5 and mCherry-Rab7, markers for early and late macropinosomes respectively (Feliciano et al., 2011; Vitelli et al., 1997). Confocal microscopy revealed the colocalization of both markers with macropinosomes (Figures 3C, D).

Actin cytoskeleton, Rac1, and cholesterol are required for macropinosome formation. To further characterize the TSL-induced macropinosomes, we used F-actin polymerization inhibitor cytochalasin D (CytD) (Nakase et al., 2004), Rac1 inhibitor EHT1864, and the cholesterol-depleting agent Methyl-β-cyclodextrin (mβCD) (Ramirez et al., 2019) to modulate their development. Accordingly, CytD significantly reduced TSL-induced macropinosome formation (Figure 3E). Both EHT1864 and mβCD markedly mitigated TSL-induced macropinocytosis in SK-BR-3 and ZR-75-1 breast cancer cells (Figures 3F, G).

The lysosomal system is integral to the macropinocytotic process, as the eventual fusion of macropinosomes with lysosomes allows the release of amino acids and molecules to support cell growth (Palm, 2019). However, TSL-induced macropinosomes, as visualized by FITC-dextran (70 kDa), did not extensively overlap with lysosomes stained with LysoTracker Red in SK-BR-3 and ZR-75-1 cells (Figure 3H). This indicates a dysfunctional macropinocytotic process that fails to properly fuse with the lysosomal system for subsequent hydrolysis or undergo recycling to the plasma membrane. It was supported by the limited colocalization of macropinosomes with the recycling endosome marker Rab11 (Figure 3I). Notably, SEPT6, which promotes macropinosome traffic to the lysosome by facilitating membrane fusion, was downregulated with TSL treatment (Figure 2D), consistent with limited macropinosome-lysosome fusion (Figure 3H). The reduction of ADP ribosylation factor 6 GTP-binding protein (ARF6) by TSL (Figure 2D), which regulates the formation of membrane protrusions mediating the recycling of macropinosomal components back to the plasma membrane (Radhakrishna et al., 1996), aligns with the lack of overlap between macropinosome and recycling endosome (Figure 3I). Instead, these vesicles converge to generate large and harmful vacuoles (Figure 2B), which typically coalesce and disrupt normal cellular processes and eventually cause methuosis, a form of macropinocytic cell death (Maltese and Overmeyer, 2014; Nara et al., 2012).

### 3.4 Tanshinlactone induces methuosis through the activation of NRF2

**FIGURE 4:**
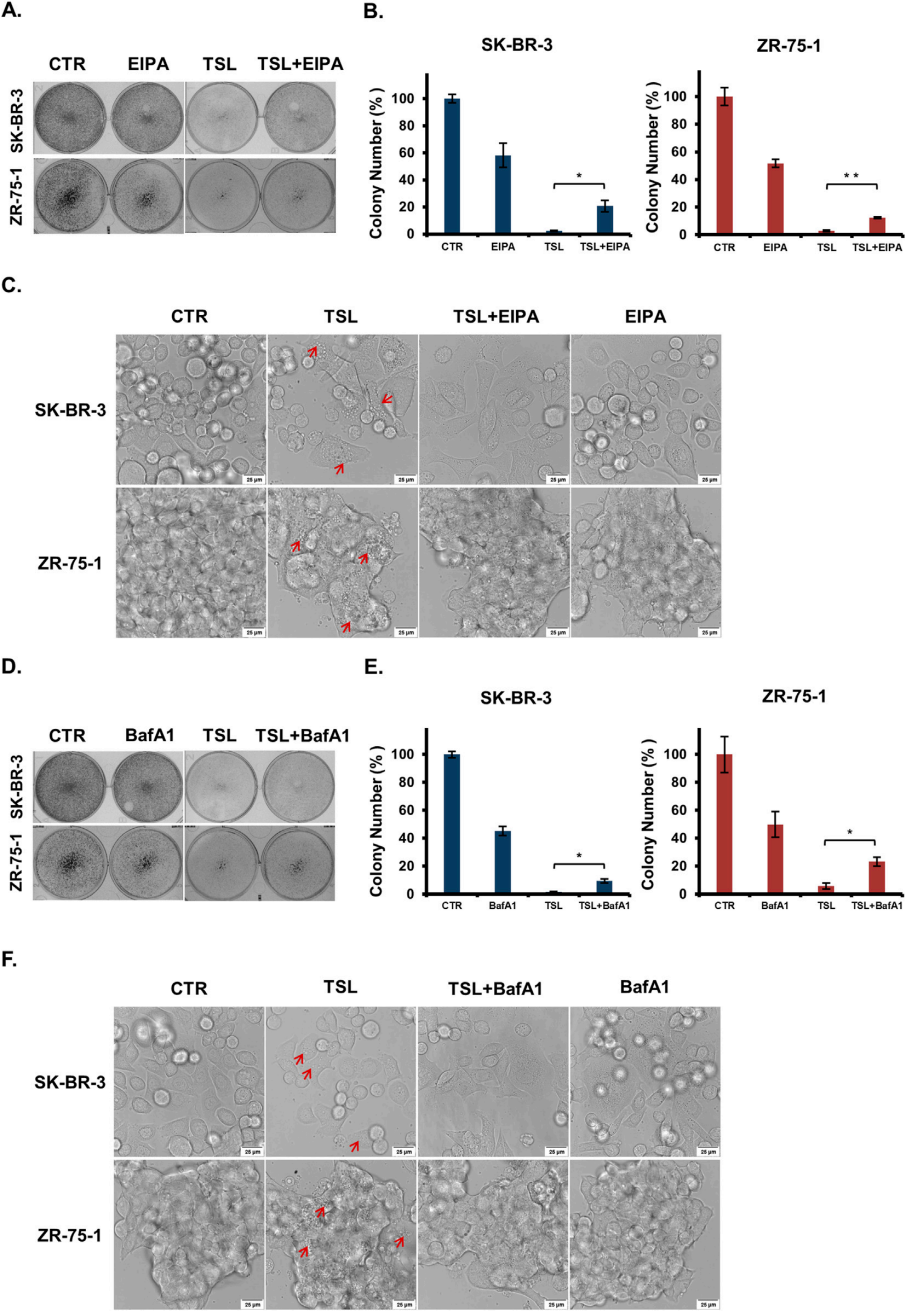
Macropinocytosis inhibitors (EIPA and BafA1) treatment abrogate tanshinlactone-induced catastrophic vacuolization and cell death in breast cancer cell lines SK-BR-3 and ZR-75-1. **(A)** Colony formation of breast cancer cell lines SK-BR-3 and ZR-75-1 after TSL (6.32 μΜ, 48 h) treatment with or without EIPA (10 μΜ, 48 h) treatment for 7–14 days. **(B)** Colony formation plates were quantified by counting colony number (n = 3). **(C)** Confocal microphotographs showing that TSL (6.32 μΜ, 24 h) induced vacuoles of SK-BR-3 and ZR-75-1 cells with or without EIPA treatment (10 μΜ, 24 h) (63×). Red arrows indicating the formation of vacuoles. The scale bar is 25 μm. **(D)** Colony formation of breast cancer cell lines SK-BR-3 and ZR-75-1 after TSL (6.32 μΜ, 48 h) treatment with or without BafA1 (100 nΜ,1 h) pretreatment for 7–14 days. **(E)** Colony formation plates were quantified by counting colony number (n = 3). **(F)** Confocal microphotographs showing that TSL (6.32 μΜ, 24 h) induced vacuoles of SK-BR-3 and ZR-75-1 cells with or without BafA1 pretreatment (100 nM, 1 h) (63×). Red arrows indicating the vacuoles. The scale bar is 25 μm. Representative images are shown (n = 3).

We next sought to verify that the cell death induced by TSL is indeed methuosis. The NHE blocker 5-(N-Ethyl-N-isopropyl)-Amiloride (EIPA) is a potent pharmacological agent known to inhibit the plasma membrane Na+/H+ exchange pump, which in turn lowers the submembranous pH and inhibits actin polymerization necessary for macropinocytosis (Koivusalo et al., 2010; West et al., 1989). TSL-induced cell death in SK-BR-3 and ZR-75–1 cells was partially mitigated by EIPA (Figures 4A, B), and cytoplasmic vacuolization was almost entirely prevented when the cells were co-treated with EIPA (Figure 4C). Similarly, bafilomycin A1 (BafA1), a specific inhibitor of the vacuolar-type H (+)-ATPase, prevents macropinosomes generation (Yoshimori et al., 1991). Pre-treatment with BafA1 effectively blocked cell death (Figures 4D, E) and the formation of cytoplasmic vacuolization (Figure 4F) induced by TSL in both SK-BR-3 and ZR-75-1 cells. On the contrary, the vacuolization could not be rescued by QVD, Lip-1, and Nec-1 (Supplementary Figures S4D–E).

**FIGURE 5:**
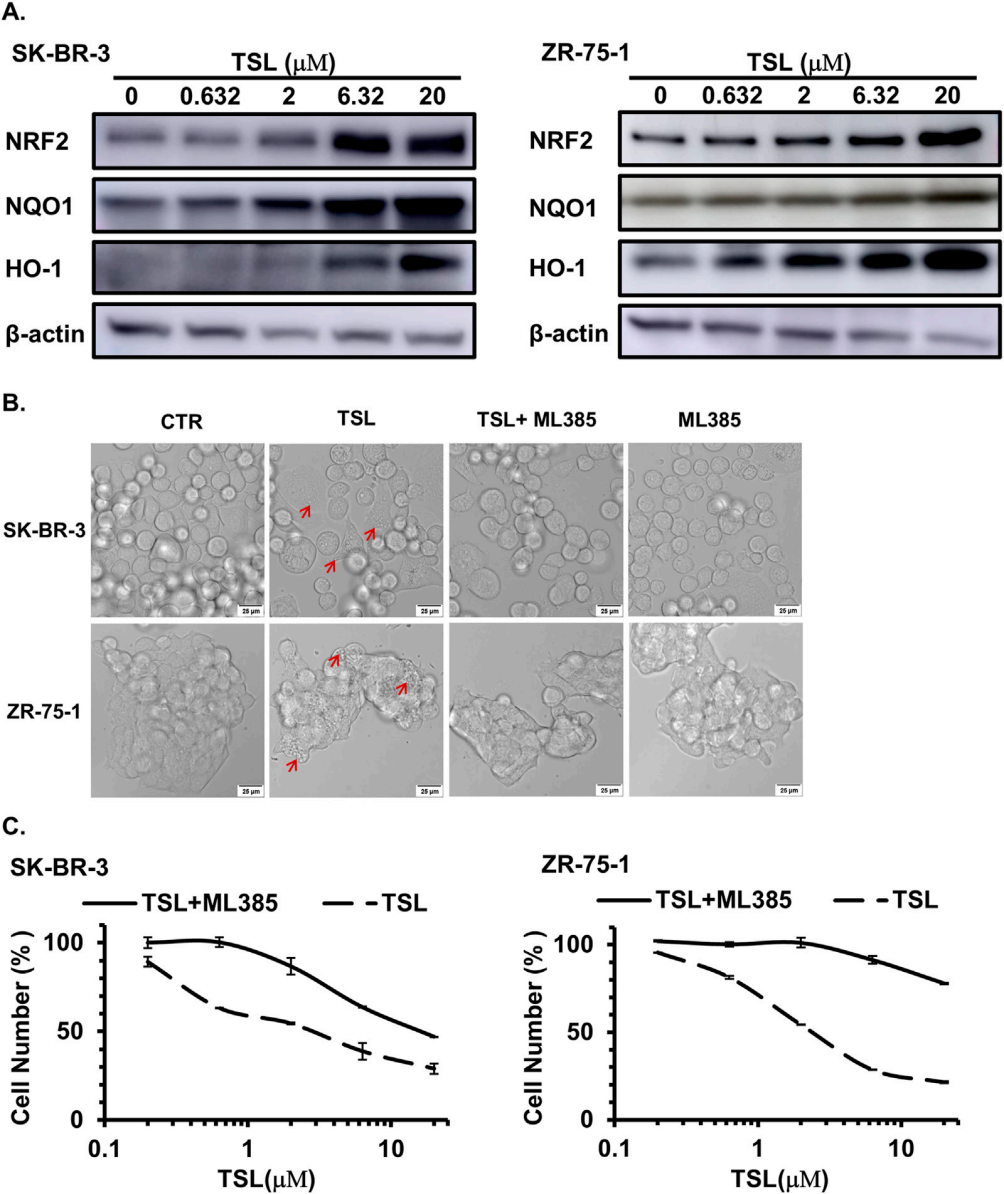
Tanshinlactone induces methuosis through the activation of NRF2 in breast cancer cell lines SK-BR-3 and ZR-75-1. **(A)** Immunoblot analyses of NRF2 and its target proteins NQO1 and HO-1 in SK-BR-3 and ZR-75-1 cell lysates treated with TSL (0, 0.632, 2, 6.32, 20 μΜ) for 48 h. Representative immunoblots are from three independent experiments (n = 3). **(B)** Confocal microphotographs showing that TSL (6.32 μΜ, 24 h) induced macropinosomes of SK-BR-3 and ZR-75-1 cells with or without NRF2 inhibitor ML385 treatment (10 μΜ, 24 h) (63×). The scale bar is 25 μm. Red arrows indicating the formation of vacuoles. **(C)** Dose effect of TSL treatment (72 h) on the proliferation of human breast cancer cell line SK-BR-3 and ZR-75-1 with or without NRF2 inhibitor ML385 treatment (10 μΜ).

The transcription factor NRF2, a recently identified regulator of macropinocytosis, is recruited to the promoter regions of macropinocytosis genes, such as EGF, CDC42, SDC1, and NHE1, in an antioxidant response element (ARE)-dependent manner (Lambies and Commisso, 2022; Su et al., 2021a). Consistently, these genes were induced by TSL treatment (Figure 2D). To determine whether TSL induces methuosis through NRF2 activation, we examined its levels in SK-BR-3 and ZR-75-1 cells. It showed that TSL significantly increased NRF2 levels, as well as its downstream effectors NQO1 and HO-1 (Figure 5A). Similar results were observed in other TSL-sensitive breast cancer cell lines MDA-MB-453, MCF7, and MDA-MB-468 (Supplementary Figures S7A–C). However, NRF2 activation was not noticeable in TSL-insensitive MDA-MB-231 cells (Supplementary Figure S7D). ML385, a specific NRF2 inhibitor, interacts with the DNA-binding domain of NRF2 and inhibits its downstream target gene expression by interfering with the binding of the NRF2 protein complex to the ARE (Singh et al., 2016; Zhang and Gordon, 2004). Cotreatment with ML385 markedly abrogated macropinosomes formation (Figure 5B) and the cell death induced by TSL (Figure 5C).

Collectively, our findings suggest that TSL activates the NRF2 pathway, promoting the formation of macropinosomes but preventing their fusion with lysosomes and the recycling process, ultimately leading to methuosis.

### 3.5 Tanshinlactone inhibits proliferation and promotes macropinocytosis in xenograft tumor explants derived from ZR-75-1

**FIGURE 6:**
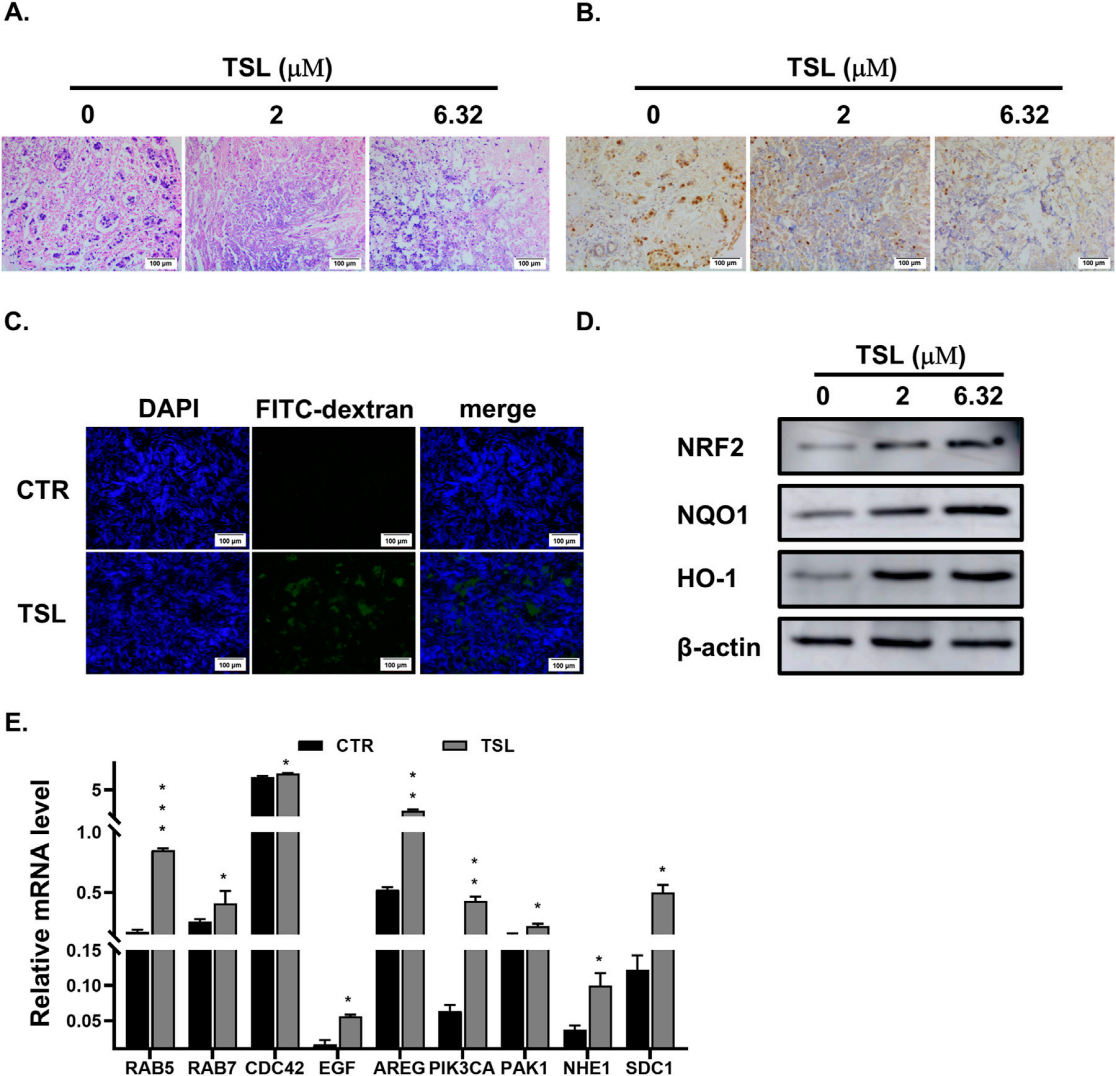
Tanshinlactone inhibits proliferation and promotes macropinocytosis in xenograft tumor explants derived from ZR-75-1. **(A)** Hematoxylin and eosin staining of the 2–3 mm^3^ sections of tumors derived from xenografted ZR-75-1 cells treated with TSL (0, 2, 6.32 μΜ) for 72 h (20×). The scale bar is 100 μm. **(B)** Immunohistochemical staining of Ki67 in the TSL-treated (0, 2, 6.32 μΜ) sections of ZR-75-1 cells derived xenograft tumors for 72 h (20×). The scale bar is 100 μm. **(C)** Visualization of macropinocytosis in ZR-75-1 derived xenograft tumor sections. Representative images from sections of ZR-75-1 xenograft tumors treated with TSL (6.32 μΜ) for 24 h stained with FITC-dextran (70 kDa) and DAPI (20×). The scale bar is 100 μm. **(D)** Immunoblot analyses of NRF2 and its target proteins NQO1 and HO-1 in ZR-75-1 derived xenograft tumor sections treated with TSL (0, 2, 6.32 μΜ) for 48 h. Representative images are from three independent experiments performed in duplicate (n = 3). **(E)** Quantitative real-time PCR analyses of mRNA levels of *RAB5, RAB7, CDC42, EGF, AREG, PIK3CA, PAK1, NHE1,* and *SDC1* in ZR-75-1 xenograft tumor sections after treatment with TSL (6.32 μΜ) for 24 h.

The induction of methuosis highlights the potential of TSL as a targeted therapeutic agent for specific breast cancer subtypes. Given the limited availability of TSL, we adopted a histoculture approach as an alternative to traditional xenograft models to evaluate the compound’s efficacy. Histoculture preserves most of the genetic and transcriptomic heterogeneity of the primary tumor tissues, providing a valuable platform for examining histological features, proliferative capacity, and molecular signaling that direct drug efficacy on solid tumors (Centenera et al., 2018; Powley et al., 2020). We proceeded to examine the effect of TSL on explants derived from ZR-75-1 xenograft tumors. Hematoxylin and eosin (HE) staining confirmed that the explanted tissue sections retained the overall morphology of invasive ductal carcinoma with high nuclear grade after 72 h of culture (Figure 6A, 0 μΜ). Notably, TSL treatment resulted in significant histological changes, with clear deviations from the control sections in terms of cellular features and overall tissue structure (Figure 6A, 2, 6.32 μΜ). Immunohistochemistry (IHC) staining of Ki67 confirmed the proliferative capacity of the cultured tissues (Figure 6B, 0 μΜ). Treatment with TSL led to a dose-dependent reduction in Ki67-positive nuclei, indicating suppressed tumor proliferation (Figure 6B, 2, 6.32 μΜ). Additionally, TSL administration was associated with increased macropinocytic uptake of FITC-dextran (70 kDa) in the tumor explants (Figure 6C), consistent with the *in vitro* observations. Immunoblotting analysis confirmed that TSL activated the NRF2 signaling pathway (Figure 6D). Correspondingly, transcriptional analysis showed that TSL upregulated NRF2-dependent genes associated with macropinocytosis in TSL-treated tumor explant sections (Figure 6E). In summary, these findings from the histoculture study provided evidence that TSL effectively inhibits proliferation and promotes NRF2-driven macropinocytosis in breast cancer explants.

### 3.6 Tanshinlactone inhibits the proliferation of SK-BR-3 cells resistant to lapatinib

**FIGURE 7:**
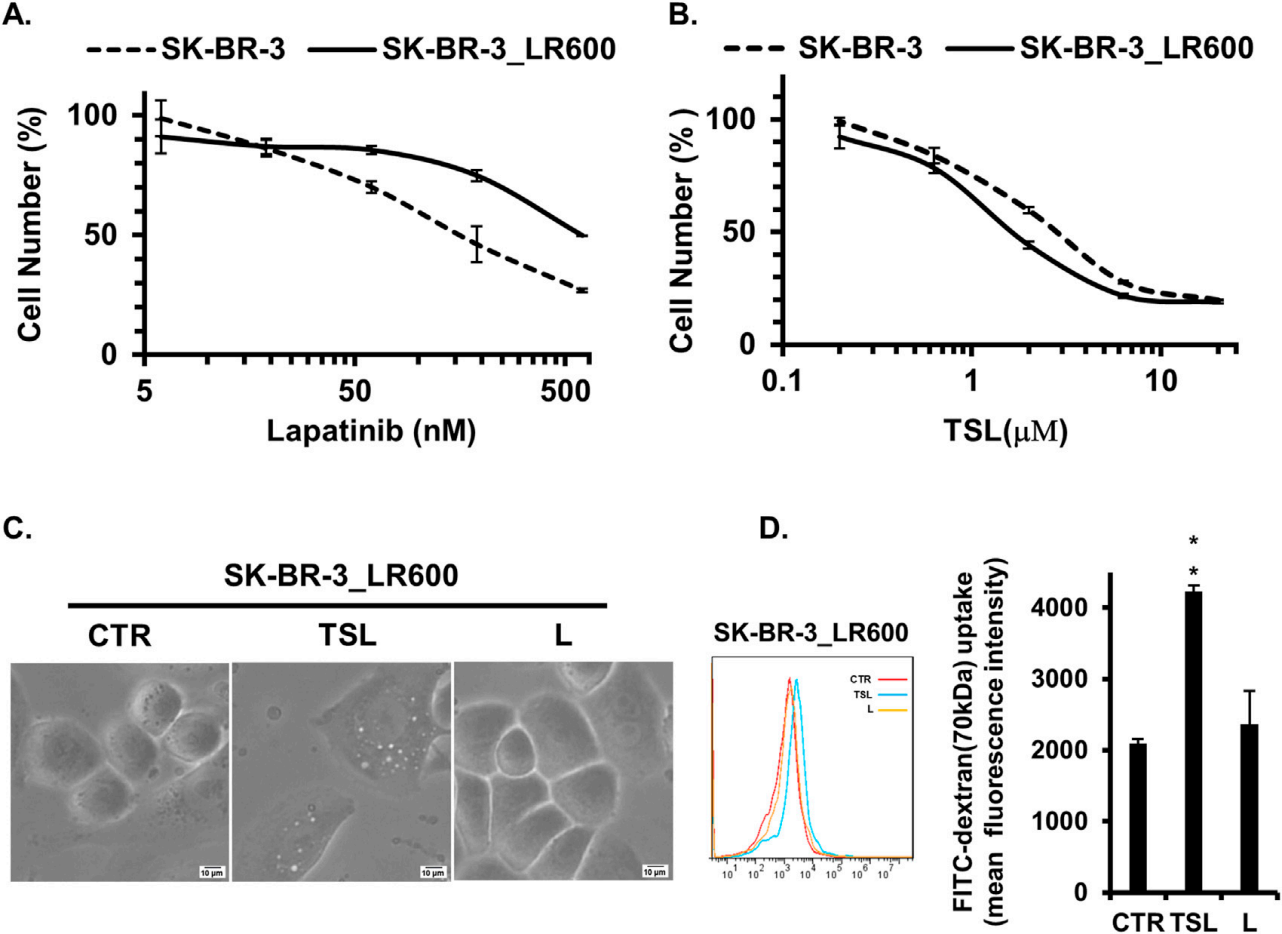
Tanshinlactone inhibits the proliferation of SK-BR-3 cells resistant to lapatinib. **(A)** Dose effect of lapatinib treatment (72 h) on the proliferation of lapatinib-resistant cell line SK-BR-3_LR600 and parental breast cancer cell line SK-BR-3. **(B)** Dose effect of TSL treatment (72 h) on the proliferation of SK-BR-3_LR600 and SK-BR-3. **(C)** Phase-contrast images showing effects on cytoplasmic vacuolization of SK-BR-3_LR600 cells following treatment of TSL (6.32 μM, 24 h) and lapatinib (600 nM, 24 h) (20×). The scale bar is 10 μm. **(D)** Quantitative FITC-dextran (70 kDa) uptake by SK-BR-3_LR600 determined by flow cytometry after treated with TSL (6.32 μM) and lapatinib (600 nM) for 24 h.

Currently, tyrosine kinase inhibitors (TKIs) are the primary targeted therapy for patients with HER2/EGFR-positive breast cancers (Howe and Brown, 2011). Lapatinib, a TKI approved by the FDA in 2007, works by competitively binding to the ATP-binding site of the HER2/EGFR tyrosine kinase (Medina and Goodin, 2008). However, resistance to lapatinib presents a significant challenge, especially in managing advanced stages of the disease where prolonged exposure to targeted therapies often leads to the development of resistance mechanisms (D'Amato et al., 2015). Given the efficiency of methuosis induction on cancer cells resistant to traditional chemotherapies, we explored the effect of TSL on lapatinib-resistant breast cancer cells. To test this, we generated lapatinib-resistant clones, SK-BR-3_LR600, from the SK-BR-3 cell line by gradually increasing the dose of lapatinib. The IC_50_ increased from 150 nM of the parental cell line to 600 nM of the SK-BR-3_LR600 cell line (Figure 7A). However, SK-BR-3_LR600 showed similar sensitivity to TSL (Figure 7B), and the IC_50_ was 1.62 μΜ, demonstrating the effectiveness of TSL on lapatinib-resistant cells. Specifically, TSL maintained its ability to induce extensive cellular vacuolation in SK-BR-3_LR600 cells (Figure 7C). Furthermore, flow cytometry quantification of dextran internalization revealed a significant increase in the uptake of FITC-dextran (70 kDa) by TSL treatment in SK-BR-3_LR600 cells compared to both DMSO and lapatinib treatment (Figure 7D). This suggests that TSL could be a potential therapeutic agent for lapatinib-resistant breast cancer cells.

## 4 Discussions

**FIGURE 8:**
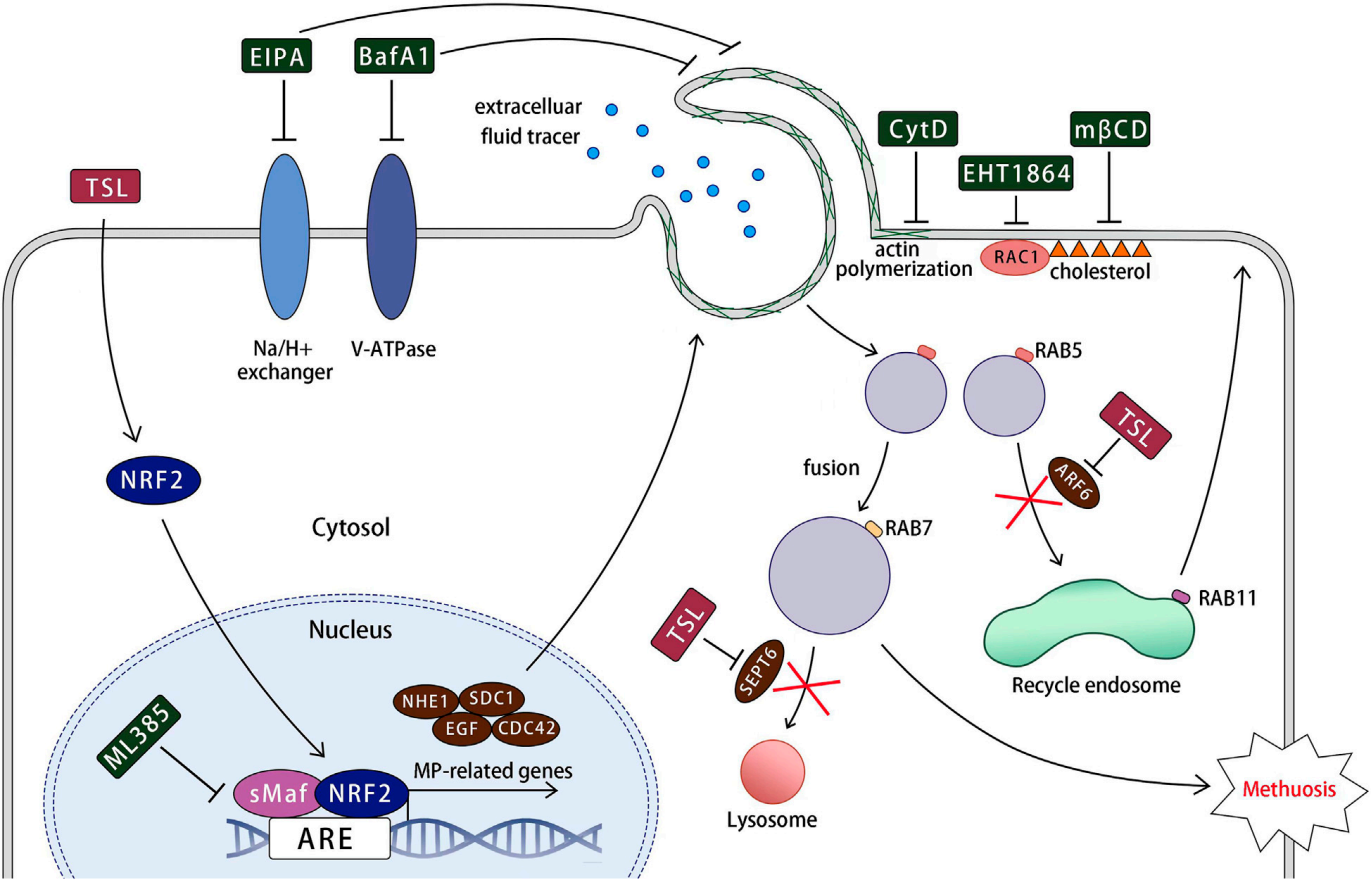
A working model for tanshinlactone-induced methuosis suggested by the present studies. TSL activates NRF2 and its target macropinocytosis-related genes (NHE1, SDC1, EGF, and CDC42), promoting lamellipodial membrane projections and macropinosome formation. This process involves regulatory factors such as Na+/H+ exchanger, V-ATPase, actin polymerization, RAC1, and cholesterol. The formed macropinosomes rapidly mature, acquiring the characteristics of both early and late endosomes (RAB5 and RAB7). These abnormal macropinosomes undergo homotypic fusion, generating large vacuoles that are unable to recycle like normal macropinosomes or fuse with lysosomes due to the downregulation of key molecular components required for fusion with recycling endosomes and lysosomes (ARF6 and SEPT6) by TSL. Eventually, the massive macropinosomes fill the cell, leading to membrane rupture.

A working model for TSL-induced methuosis suggested by the present studies is shown in Figure 8. Our study highlights that TSL selectively inhibits the proliferation of ER+ or HER2+/EGFR+ breast cancer cell lines, but not other cancer or normal cells (Figures 1A–E, Supplementary Figures S1A–I). This selectivity is due to the induction of catastrophic vacuolization, a process characterized by the formation of numerous vesicles within the cells. Predominantly, these vesicles originate from macropinosomes, as evidenced by the uptake of fluorescent markers such as FITC-dextran (70 kDa) (Figures 3A, B) and lucifer yellow (Supplementary Figures S6A–D). Importantly, the induction of macropinosomes and subsequent cell death triggered by TSL can be effectively reversed by the macropinocytosis inhibitors, EIPA and BafA1 (Figures 4A–F). This provides strong evidence that the mechanism of cell death is directly linked to macropinocytosis induction. This unique form of cell death, characterized by excessive vesicle formation, has been labeled “methuosis”, representing a distinct and seldom-reported mode of non-apoptotic cell death (Maltese and Overmeyer, 2014). Furthermore, this uniqueness of TSL-induced cell death contributes to its efficacy on lapatinib-resistant cells (Figure 7B), highlighting its potential as a powerful adjuvant that can overcome drug resistance, a major challenge in cancer treatment. This could be due to methuosis is not cross-resistant to apoptosis, as many other non-apoptotic forms of cell death. Therefore, the induction of methuosis by TSL treatment represents a novel strategy in cancer therapeutics, especially considering its specificity towards certain breast cancer subtypes. AS1411, a nucleolin targeting aptamer that had been tested as an anticancer agent in Phase II clinical trials, could stimulate macropinocytosis to promote drug uptake in cancer cells but not nonmalignant cells (Reyes-Reyes et al., 2010), suggesting the potential application of macropinocytosis process in drug delivery for cancer treatment. Considering TSL as an inducer of macropinocytosis, the unique mechanism would be expected to improve drug delivery specifically in cancer therapy and explore the synergistic effects of TSL with existing therapeutic approaches.

This unusual form of cell death has been observed in certain cancer cells treated with certain small molecules. MIPP and MOMIPP have been reported to cause extreme vacuolization derived from macropinosomes and induced cell death in glioblastoma cells (Overmeyer et al., 2011; Robinson et al., 2012). Recent studies suggest possible mechanisms for these indolyl-pyridinyl-propenones (IPPs), such as inhibition of the phosphatidylinositol-3-phosphate 5-kinase (PIKfyve) (Cho et al., 2018) and activation of the JNK1/2 stress kinase pathway (Li et al., 2019). Methamphetamine causes cytotoxicity by inducing macropinocytosis through the activation of Ras and Rac1 and lysosomal dysfunction in human neuroblastoma cells (Nara et al., 2012). Dual inhibitors of mTORC1/mTORC2, such as OSI-027, PP242, MLN0128, and Torin 1, induce catastrophic vacuolization in tumor cell lines from various tissues (Srivastava et al., 2019). Other drugs promoting methuosis-like cell death in cancer cell lines, such as vacquinol-1 in glioblastomas (Sander et al., 2017), silmitasertib in colorectal cancer cells (Silva-Pavez et al., 2019), jaspine B in gastric cancer cells (Cingolani et al., 2017), and tubeimoside-1 in multiple colorectal cancer cell lines (Gong et al., 2018), are being investigated. Considering methuosis as a distinctive form of cell death and its selective occurrence in certain cancer cell lines, the targeted development of anticancer therapies exploiting this mechanism holds immense therapeutic promise (Overmeyer et al., 2011; Sun et al., 2017). Based on our examination of several cancer cell lines, it appears that the ability of TSL to induce methuosis is limited to ER+ and HER2+/EGFR+ breast cancer cells. Notably, TSL demonstrates minimal toxicity to normal cells even at high doses, compared to other methuosis inducers, underscoring its potential as a targeted therapy with a favorable safety profile.

This study reveals a novel link between NRF2 activation and catastrophic macropinocytosis in breast cancer cell lines. Our research pioneers the concept that NRF2 activation by TSL enables the transcriptional activation of macropinocytosis-related genes (Figures 5A, 2D), leading to cell death through massive macropinosomes polymerization. This was evidenced by the fact that ML385, an inhibitor of NRF2, was able to rescue cell death and macropinosome formation induced by TSL treatment (Figures 5B,C). Previously, Hua Su et al. reported NRF2-driven enhancement of macropinocytotic flux in autophagy-inhibited, hypoxic, or oxidatively stressed cancer cells, allowing them to import extracellular proteins to support their bioenergetic demands (Su et al., 2021b). However, NRF2-induced macropinocytosis has a controversial effect on the fate of tumor cells (Su et al., 2021a), underscoring the complexity and plasticity of cellular responses to stress. Moreover, oncogenic RAS, its aberrant expression and activation, also contributes to hyperactive macropinocytosis and diverse cellular outcomes. Cosimo Commisso found that cancers driven by oncogenic RAS utilize heightened macropinocytosis to augment their nutrient supply and maintain proliferation (Commisso et al., 2013). Simultaneously, Jean H. Overmeyer implicated similar forms of cell death associated with active RAS driving excessive macropinocytosis in Glioblastoma Cells (Overmeyer et al., 2008). Given that macropinocytosis is a hallmark of cancer cells carrying oncogenes, further investigation into these intricate signaling pathways has the potential to unearth additional chemical and physiological stimuli responsible for initiating this unconventional mode of cell death, thereby enhancing our understanding and potentially opening new avenues for cancer treatment and therapeutic intervention.

Macropinocytosis is defined as an actin-dependent but coat- and dynamin-independent endocytic uptake process, which brings large fluid-filled macropinosomes into the cell interior (Doherty and McMahon, 2009). Functional macropinosomes navigate two principal pathways upon formation: they are either directed toward lysosomes for the degradation of their cargo or recycled back to the plasma membrane to ensure a continuous supply of nutrients, facilitate membrane remodeling, and maintain cellular homeostasis (Donaldson, 2019). When methuosis happens, the normal endosomal trafficking pathway is disrupted instead of coalescing to form giant vacuoles and subsequent cell death (Maltese and Overmeyer, 2014). The TSL-induced macropinosomes acquire characteristics of early and late endosomes, as indicated by the presence of Rab5 and Rab7 proteins on the membrane of macropinosomes, respectively (Figures 3C, D). However, these structures do not align with the characteristics of recycled endosomes (Figure 3I), or lysosomal compartments (Figure 3H). Ultimately, the displacement of much of the cytoplasmic space by the accumulated vacuoles is accompanied by the rupture of the cancer cells (Figures 2A–C). In the recycling pathway, maturing macropinosomes integrate with sorting early endosomes and recycling endosomes, eventually returning to the plasma membrane for reuse (Banushi et al., 2023). TSL-induced macropinosomes, which are positive for Rab5 and negative for Rab11, do not acquire the characteristics of recycling endosomes. This observation can be elucidated by postulating that these newly formed macropinosomes swiftly progress through maturation stages, bypassing the intermediate recycling phase. Instead, they develop traits associated with late endosomes, presumably enroute to a terminal fusion event. Rab7 plays an important role in facilitating late endosome-lysosome fusion (Bucci et al., 2000; Luzio et al., 2010). It remains unclear why the TSL-induced Rab7+ vacuoles are capable of undergoing fusion with each other yet fail to merge with lysosomes. A plausible explanation for this phenomenon is that TSL treatment swiftly activates the transcription factor NRF2, which in turn accelerates the expression of genes associated with macropinocytosis. This heightened pace of vacuole formation, coupled with rapid vacuole fusion, overwhelms the cells’ capacity to manage these structures effectively. Instead of following the standard endosomal trafficking route, these vacuoles circumvent the necessary steps for acquiring crucial proteins that facilitate their proper docking and fusion with lysosomes. Consequently, the lack of these key proteins disrupts the homotypic and heterotypic vesicular interactions (Luzio et al., 2009; Wang et al., 2011), preventing the degradation of vacuolar contents and ultimately resulting in the accumulation of non-functional vesicles. This dysregulated process, driven by accelerated macropinocytosis, contributes to abnormal cellular drinking and, ultimately, the rupture of cancer cells. This phenomenon can also be explained that chronic or excessive NRF2 activation may interfere with normal metabolic pathways (He et al., 2020), including those related to lysosomal biogenesis and functional maintenance (Kim et al., 2024; Ong et al., 2023), indirectly affecting lysosomal activity.

This study focused on the *in vitro* anti-breast cancer evaluation of TSL, providing initial insights into its pharmacological properties. However, further validation through *in vivo* studies is essential. We evaluated the drug-like properties of TSL using a computational tool. The molecular weight of TSL is 264.28, the number of hydrogen bond donors is 0, the number of hydrogen bond acceptors is 3, the QP log P for octanol/water is 3.208, and the number of rotatable bonds is 0, which meets the five principles of drug-like properties. All 15 molecular property indicators of TSL were within the reference ranges of 95% of known drugs, underscoring its potential for good drugability (Table 3). Predictions of the ADME properties showed that its QP log P for octanol/water, QP log S for aqueous solubility, and CIQP log S are both within a reasonable range, indicating that TSL has good solubility. The high QPPCaco, QPPMDCK, and Human Oral Absorption imply rapid intestinal uptake, high renal permeability, and efficient oral bioavailability (Table 3). Therefore, the various indicators predicted by computer virtualization indicate that TSL has good drug potential. Additional pharmacokinetic assessments are needed to understand TSL’s bioavailability, ADME (absorption, distribution, metabolism, and excretion) properties, and safety profile. TSL has received less research attention compared to other constituents of *Salvia miltiorrhiza* (Nwafor et al., 2021; Zhang et al., 2012). However, given its pharmacological activities and the efficacy of diterpene lactone compounds, TSL shows potential as a promising precursor compound.

**TABLE 3:** Molecular and ADME properties of tanshinlactone predicted by the QikProp.

	Indicators	Descriptors	Range
Molecular properties	Molecular weight	264.28	130.0–725.0
	Dipole moment (D)	4.803	1.0–12.5
	Total SASA	495.194	300.0–1,000.0
	Hydrophobic SASA	177.915	0.0–750.0
	Hydrophilic SASA	54.418	7.0–330.0
	Carbon Pi SASA	262.862	0.0–450.0
	Weakly polar SASA	0	0.0–175.0
	Molecular volume (A^3)	834.211	500.0–2000.0
	vdW polar SA (PSA)	45.358	7.0–200.0
	No.of rotatable bonds	0	0.0–15.0
	as Donor-hydrogen bonds	0	0.0–6.0
	as Acceptor-hydrogen bonds	3	2.0–20.0
	Globularity (sphere = 1)	0.8,654,461	0.75–0.95
	Ionization potential (eV)	8.691	7.9–10.5
	Electron affinity (eV)	1.26	−0.9–1.7
ADME properties	QPlogP for octanol/water	3.208	−2.0–6.5
	QPlogS for aqueous solubility	−4.03	−6.5–0.5
	CIQPlogS	−4.313	−6.5–0.5
	QPPCaco	3,018.939	<25 poor; >500 great
	QPPMDCK	1,633.111	<25 poor; >500 great
	% Human oral absorption	100	<25 poor; >80 great
	Human oral absorption	3	1 low; 2 moderate; 3 high

The exact molecular mechanisms underlying catastrophic vacuolization from macropinosomes are not fully understood but are believed to involve dysregulation of member trafficking. Distinct from apoptosis, this mode of cell death lacks established biomarkers or classic targets for definition, marking it as an emerging field in research that remains largely unexplored. Unraveling the precise molecular targets is crucial for understanding the selectivity of TSL’s action towards these specific subtypes of breast cancer cells and distinguishing why it does not exert similar effects on other cell types. The question of its specificity raises intriguing possibilities about the involvement of signaling pathways or receptors uniquely dysregulated or overexpressed in ER+ and HER2+/EGFR+ breast cancer cells. It may interact with a regulatory component in the NRF2 pathway, or perhaps modulate receptor tyrosine kinase signaling specifically activated in these cell types. Alternatively, TSL could be exploiting a vulnerability inherent to the enhanced endocytic and metabolic demands of these cancer cells. Future research should focus on identifying specific molecular targets of TSL, thereby increasing the chances of discovering novel druggable proteins for breast cancer therapeutics. The advancement of chemical proteomics has significantly facilitated target identification in drug discovery (Ha et al., 2021). Techniques such as Cellular Thermal Shift Assays coupled with Mass Spectrometry (CETSA-MS) can monitor changes in protein stability upon binding to TSL, helping identify direct targets. Affinity Purification-Mass Spectrometry (AP-MS) is another viable method for identifying proteins that interact with TSL.

Neo-tanshinlactone (NTSL), an isomer of tanshinlactone (TSL), is also derived from *Salvia miltiorrhiza*, commonly known as Danshen, a plant utilized in traditional Chinese medicine. Despite their similar names and shared selective inhibitory effects on certain breast cancer cell lines, these two lactones exhibit distinct chemical structures and potentially divergent biological activities. NTSL selectively inhibits the proliferation of ER+ breast cancer cell lines, such as MCF7 and ZR-75-1, through transcriptional down-regulation of estrogen receptor alpha (Lin et al., 2016). In contrast to TSL, which induces catastrophic macropinocytosis (methuosis) leading to cell death in ER+, HER2+/EGFR+ breast cancer cell lines, no cytoplasmic vacuoles were observed in NTSL-treated cells (data not shown). Both TSL and NTSL belong to the class of labdane diterpenes, featuring a lactone ring and a long hydrocarbon chain with a characteristic labdane skeleton. However, they differ in the specific arrangement of functional groups and the configuration of their rings. Specifically, the hybridization sites of their dioxatetracyclic rings vary, with NTSL being 12,17-dioxatetracyclo and TSL being 12,16-dioxatetracyclo (Wang et al., 2004). These structural differences underpin their distinct biological activities. Further experimental research is warranted to determine whether the differential activity between these two lactones is attributable to the specific functional groups present in their structures.

Currently, TSL is primarily obtained through plant extraction, with no readily available synthetic route. Additionally, the yield from plant extraction is also very low (Luo et al., 1986; Xu et al., 2006). A significant limitation of our study is the scarcity of TSL for *in vivo* experimentation, which poses a substantial barrier to advancing its clinical translation and fully realizing its therapeutic potential. To facilitate a more comprehensive assessment of both the efficacy and safety profile of TSL using *in vivo* models, it is imperative to either address the issue of authenticity of Chinese medicine or devise a practical synthetic pathway for ensuring a consistent and sufficient supply of high-quality TSL. We hope our study will promote interest in this compound and stimulate efforts towards its synthesis.

## Data Availability

The original contributions presented in the study are included in the article/Supplementary Material, further inquiries can be directed to the corresponding author.
