# Supplementary material for: Tanshinlactone triggers methuosis in breast cancer cells via NRF2 activation
Source: Front Pharmacol. 2025 Jan 21;15:1534217. doi: 10.3389/fphar.2024.1534217 (PMC11790599; doi:10.3389/fphar.2024.1534217)
Supplement: Supplementary file 2 [file DataSheet1.docx]

*Supplementary Materials*

Supplementary Figure 1


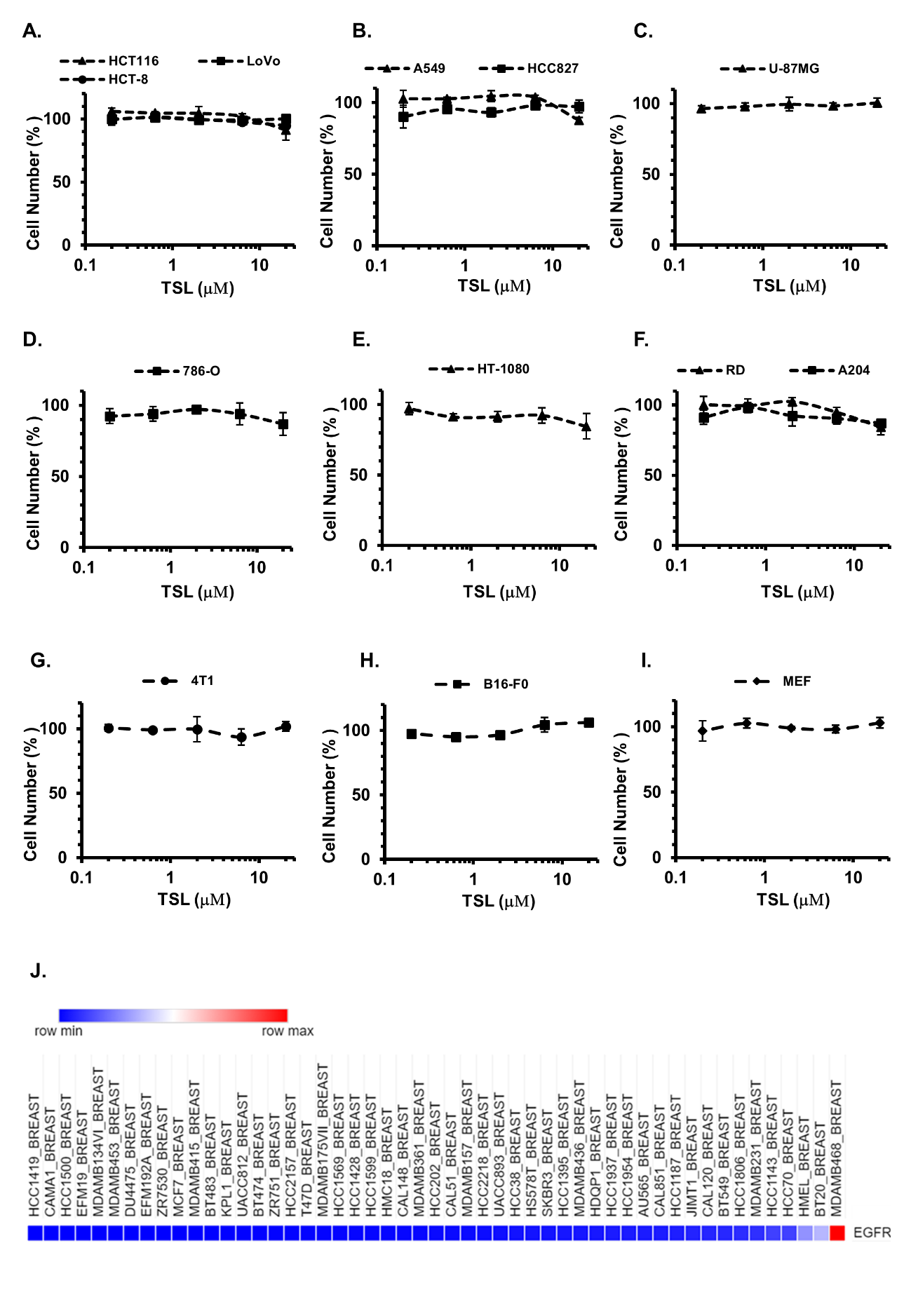


Supplementary Figure 1. Tanshinlactone does not affect other types of cancer cells and non-cancer cells. human colorectal cancer cell lines, human lung cancer cell lines, human glioblastoma cell lines, human renal carcinoma cell lines, human fibrosarcoma cell lines, human rhabdomyosarcoma cell lines, murine breast cancer cell lines, murine melanoma cell lines, and mouse embryo fibroblast were used to determine the growth inhibition effect of TSL. (A) Dose effect of TSL treatment (72 h) on the proliferation of human colorectal cancer cell lines (HCT116, LoVo, and HCT-8). (B) Dose effect of TSL treatment (72 h) on the proliferation of human lung cancer cell lines (A549 and HCC827). (C) Dose effect of TSL treatment (72 h) on the proliferation of human glioblastoma cell lines (U-87MG). (D) Dose effect of TSL treatment (72 h) on the proliferation of human renal carcinoma cell lines (786-O). (E) Dose effect of TSL treatment (72 h) on the proliferation of human fibrosarcoma cell lines (HT-1080). (F) Dose effect of TSL treatment (72 h) on the proliferation of human rhabdomyosarcoma cell lines (RD and A204). (G) Dose effect of TSL treatment (72 h) on the proliferation of murine breast cancer cell lines (4T1). (H) Dose effect of TSL treatment (72 h) on the proliferation of murine melanoma cell lines (B16-F0). (I) Dose effect of TSL treatment (72 h) on the proliferation of mouse embryo fibroblast (MEF). The cell number at each TSL concentration is represented as a percentage of control (no TSL treatment). Average values are from three independent experiments performed in duplicate (n = 3). Data are shown as mean ± SD. (J) Relative mRNA expression levels (log2) of EGFR in human breast cancer cell lines from Cancer Cell Line Encyclopedia (CCLE) database. The heatmap represents color-coded expression levels of differentially expressed EGFR in human breast cancer cell lines. The color scale ranges from saturated blue for the minimum to saturated red for the maximum.

Supplementary Figure 2


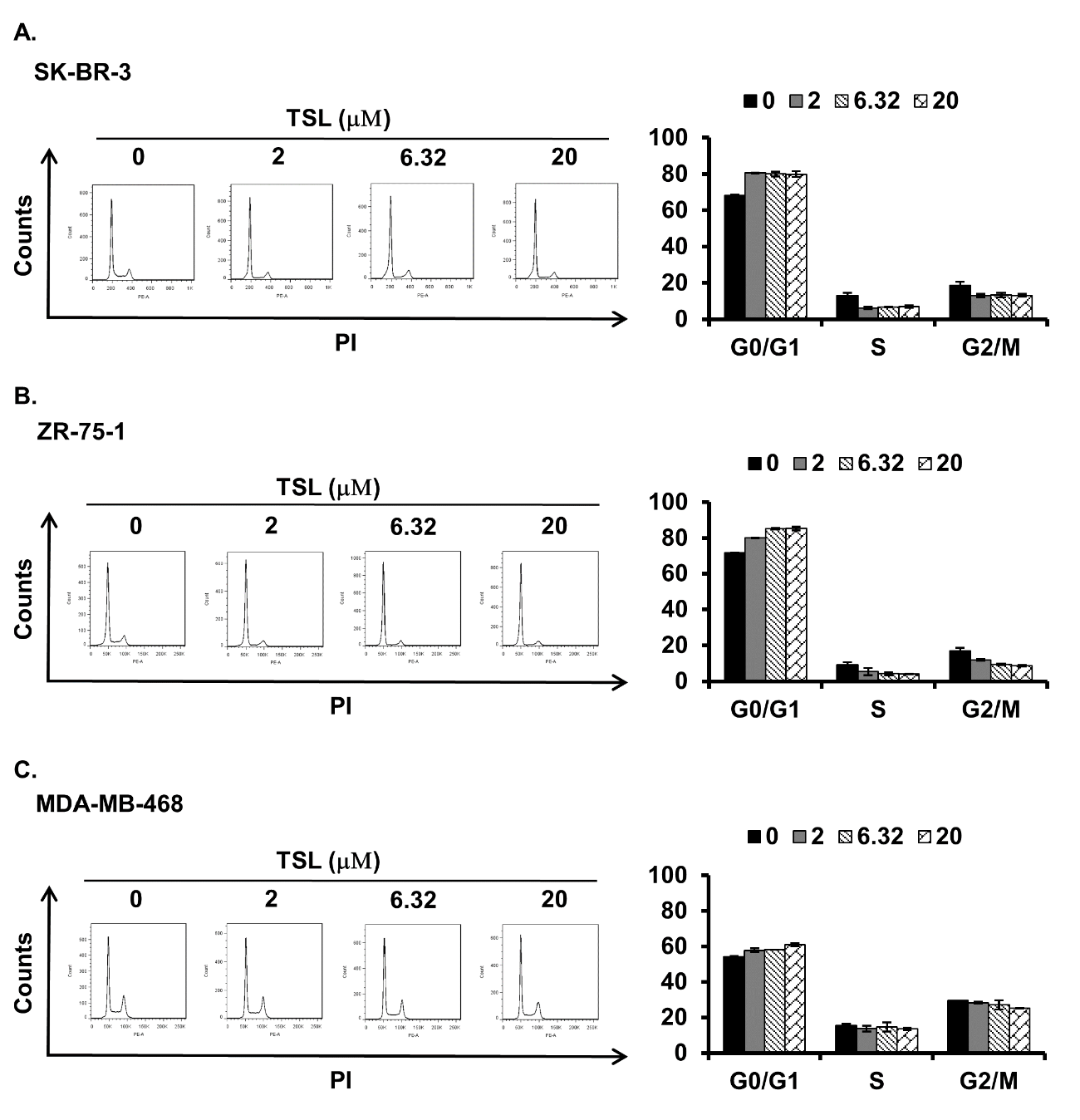


Supplementary Figure 2. Tanshinlactone does not obviously change the cell cycle distribution of SK-BR-3, ZR-75-1, and MDA-MB-468 cells. Breast cancer cell lines SK-BR-3, ZR-75-1, and MDA-MB-468 were treated with TSL (0, 2, 6.32, 20 μM) for 48 h. Representative cell cycle distributions analyzed by flow cytometry. Percentages of the total cell population in the three different phases of cell cycle (G0/G1, S, and G2/M) were determined using FlowJo software. Average values are from three independent experiments (n = 3). Data are shown as mean ± SD. (A) Cell cycle distribution of SK-BR-3. (B) Cell cycle distribution of ZR-75-1. (C) Cell cycle distribution of MDA-MB-468.

Supplementary Figure 3


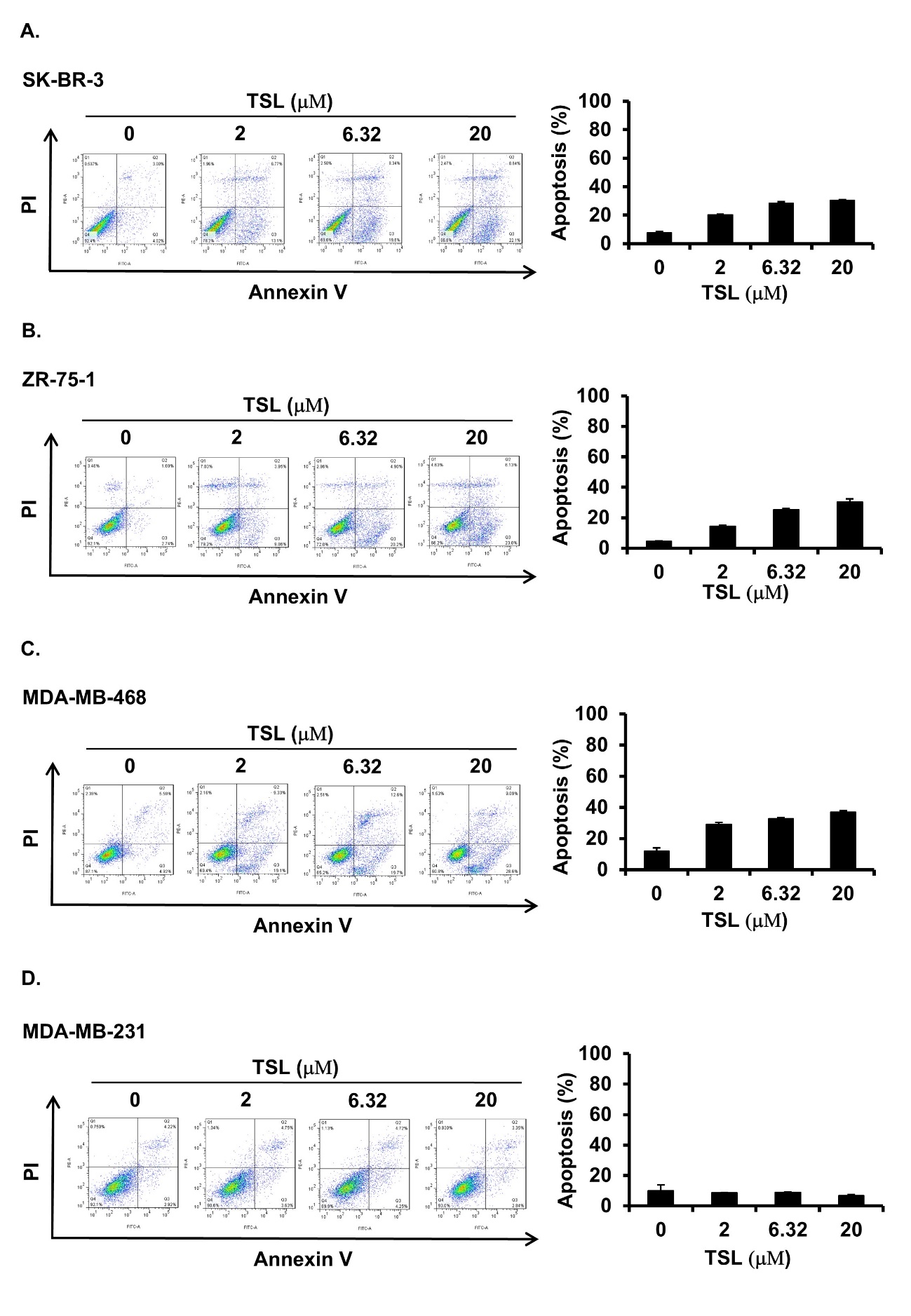


Supplementary Figure 3. Tanshinlactone minorly induces apoptosis in SK-BR-3, ZR-75-1, and MDA-MB-468 compared to MDA-MB-231 cells. Breast cancer cell lines SK-BR-3, ZR-75-1, MDA-MB-468, and MDA-MB-231 were treated with TSL (0, 2, 6.32, 20 μM) for 48 h. Representative contour diagrams of FITC Annexin V/PI flow cytometry analysis of cells. Fractions of apoptotic cells were quantified by FlowJo. Average values are from three independent experiments (n = 3). Data are shown as mean ± SD. (A) Cell apoptosis analysis of SK-BR-3. (B) Cell apoptosis analysis of ZR-75-1. (C) Cell apoptosis analysis of MDA-MB-468. (D) Cell apoptosis analysis of MDA-MB-231.

Supplementary Figure 4


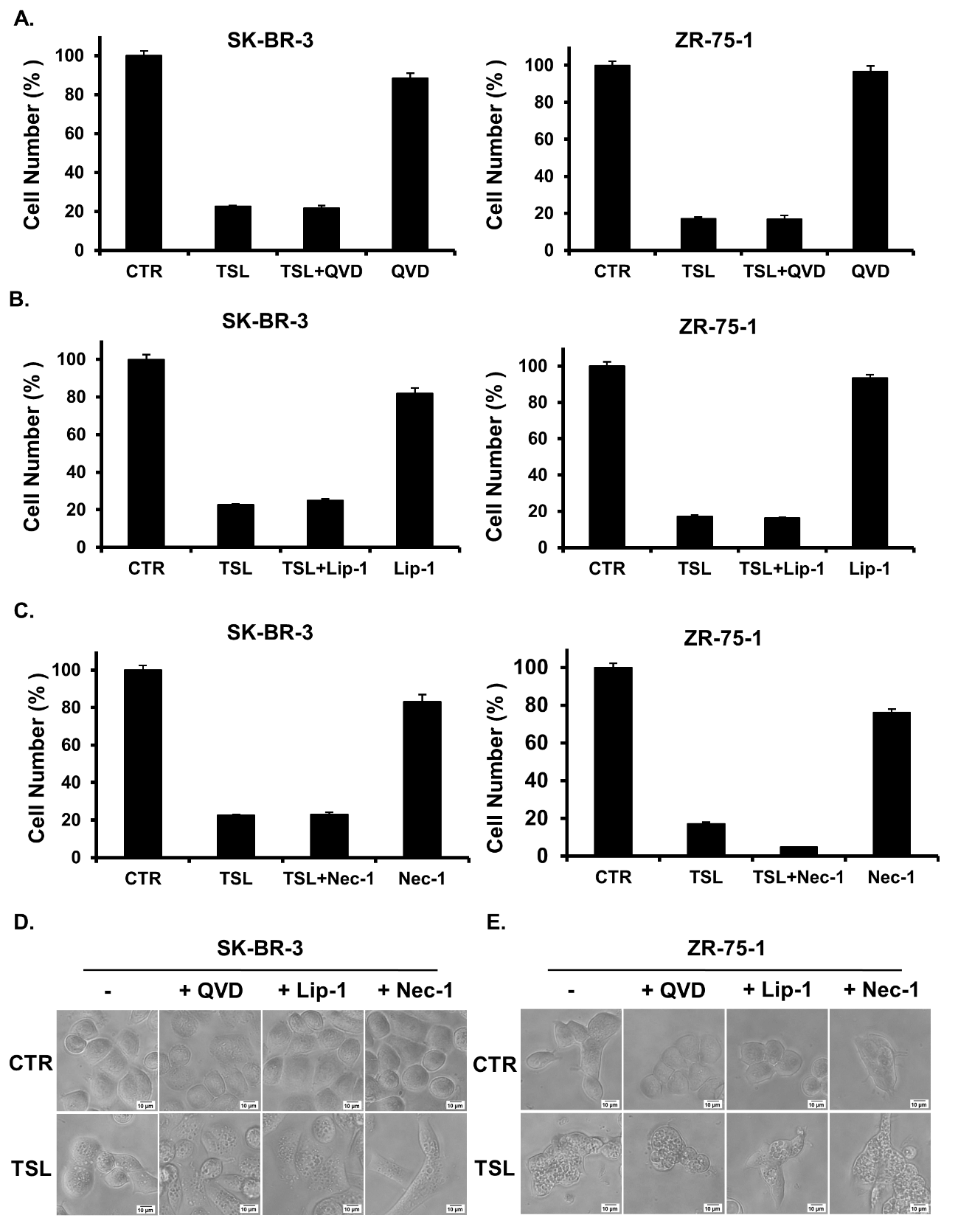


Supplementary Figure 4. Apoptosis inhibitor Q-VD-OPh, ferroptosis inhibitor liproxstatin-1, and necroptosis inhibitor necrostatin-1 treatment cannot rescue tanshinlactone-induced vacuolization and cell death in breast cancer cell lines SK-BR-3 and ZR-75-1. (A) Dose effect of TSL treatment (6.32 μM, 72 h) on the proliferation of human breast cancer cell line SK-BR-3 and ZR-75-1 with or without apoptosis inhibitor Q-VD-OPh treatment (50 μM). (B) Dose effect of TSL treatment (6.32 μM, 72 h) on the proliferation of human breast cancer cell line SK-BR-3 and ZR-75-1 with or without ferroptosis inhibitor liproxstatin-1 treatment (5 μM). (C) Dose effect of TSL treatment (6.32 μM, 72 h) on the proliferation of human breast cancer cell line SK-BR-3 and ZR-75-1 with or without necroptosis inhibitor necrostatin-1 treatment (10 μM). The experiments shown here were repeated (n=3). Data are shown as mean ± SD. (D) Phase-contrast images showing TSL (6.32 μM, 24 h) induced vacuoles of SK-BR-3 cells with or without apoptosis inhibitor Q-VD-OPh (50 μM), ferroptosis inhibitor liproxstatin-1 (5 μM) and necroptosis inhibitor necrostatin-1 (10 μM) treatment (20×). (E) Phase-contrast images showing TSL (6.32 μM, 24 h) induced vacuoles of ZR-75-1 cells with or without apoptosis inhibitor Q-VD-OPh (50 μM), ferroptosis inhibitor liproxstatin-1 (5 μM) and necroptosis inhibitor necrostatin-1 (10 μM) treatment (20×). Representative images are shown (n = 3). The scale bar is 10 μm.

Supplementary Figure 5


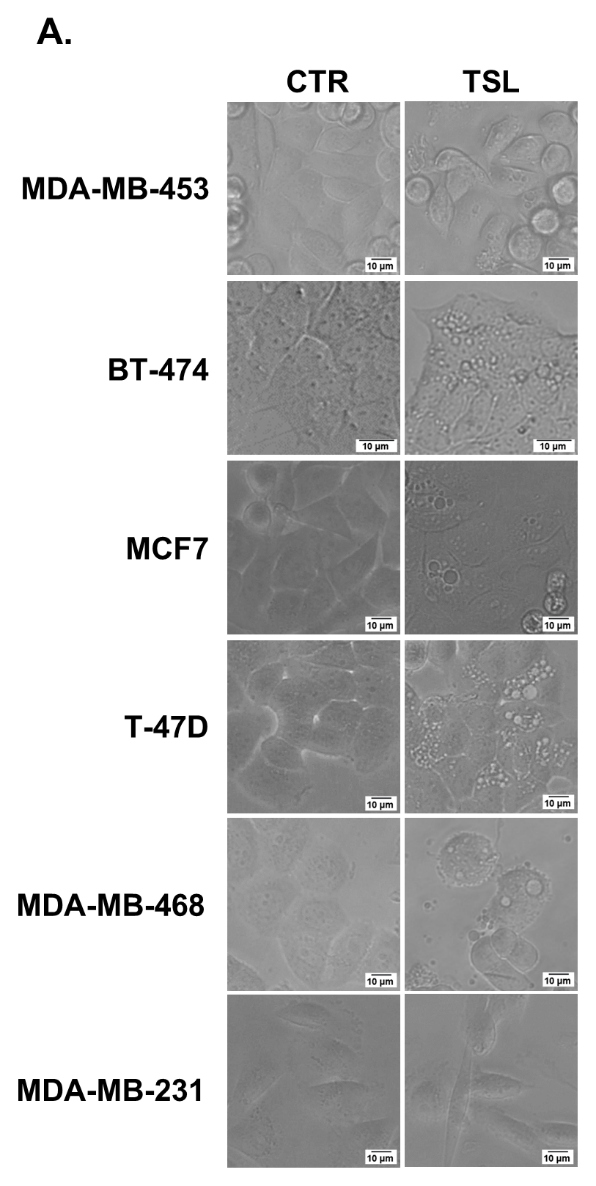


Supplementary Figure 5. Tanshinlactone induces cytoplasmic vacuolization in other breast cancer cell lines MDA-MB-453, BT-474, MCF7, T-47D, and MDA-MB-468 compared to MDA-MB-231. (A) Phase-contrast images showing effects of TSL (6.32 μM, 24 h) on cytoplasmic vacuolization in MDA-MB-453, BT-474, MCF7, T-47D, MDA-MB-468 compared to MDA-MB-231 cells (20×). These experiments represent one of three biological replicates. The scale bar is 10 μm.

Supplementary Figure 6


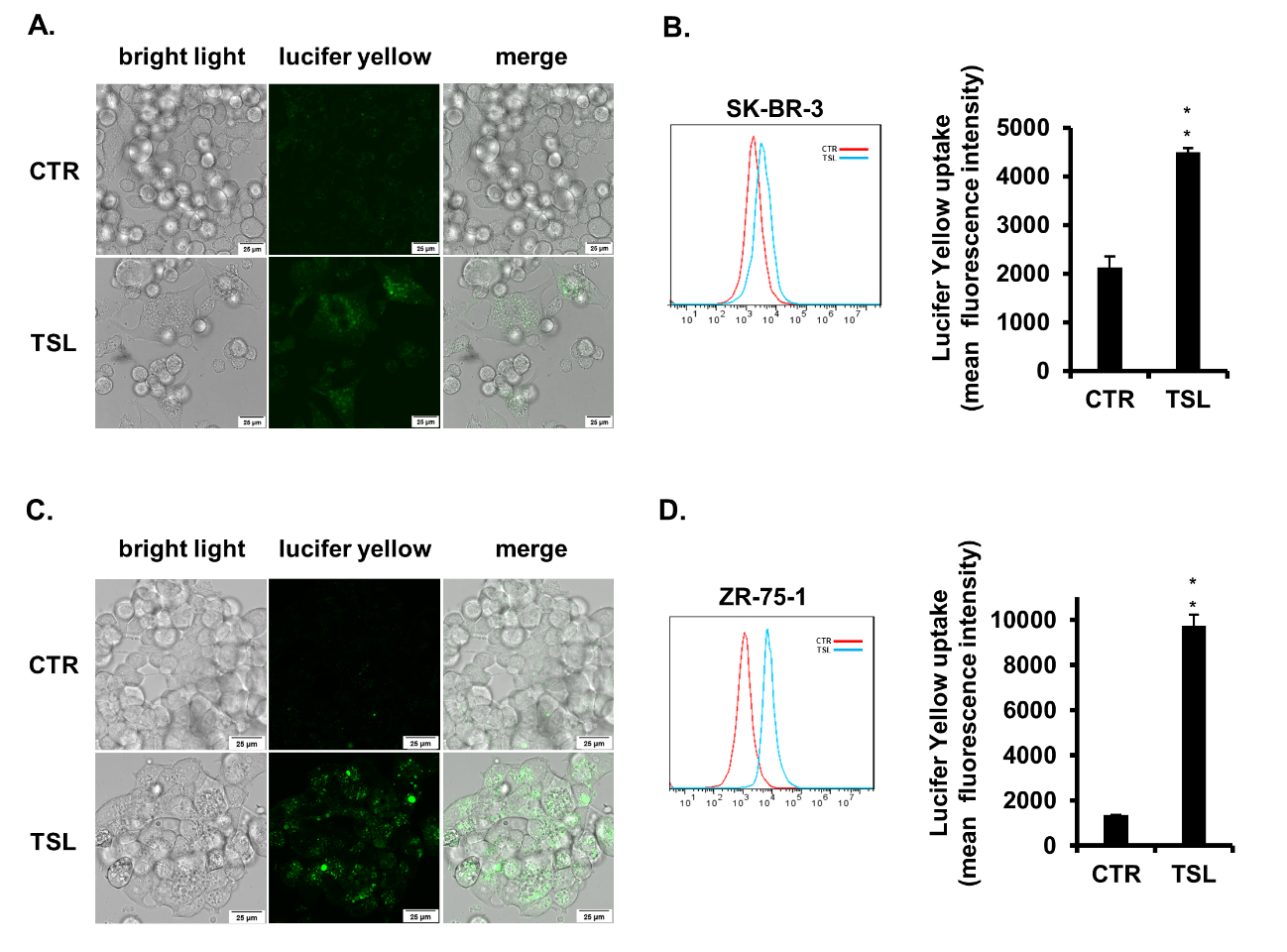


Supplementary Figure 6. Tanshinlactone-induced cytoplasmic vacuoles in breast cancer cell lines SK-BR-3 and ZR-75-1 are positive for lucifer yellow. (A) Overlay of bright field microphotographs on lucifer yellow accumulation in untreated and TSL (6.32 μM) treated SK-BR-3 cells at 24 h (63×). Most of the vacuoles were positive for lucifer yellow. (B) Quantitative lucifer yellow uptake by SK-BR-3 determined by flow cytometry after untreated and treated with TSL (6.32 μM) for 24 h. (C) Overlay of bright field microphotographs on lucifer yellow accumulation in untreated and TSL (6.32 μM) treated ZR-75-1 cells at 24 h (40×, zoom 2). Most of the vacuoles were positive for lucifer yellow. (D) Quantitative lucifer yellow uptake by ZR-75-1 determined by flow cytometry after untreated and treated with TSL (6.32 μM) for 24 h. Representative images are shown (n = 3). The scale bar is 25 μm. Average values are from three independent experiments performed in duplicate (n = 3). Data are shown as mean ± SD. P-values determined by Student’s t-test compared to control. *P < 0.05; **P < 0.01; ***P < 0.001.

Supplementary Figure 7


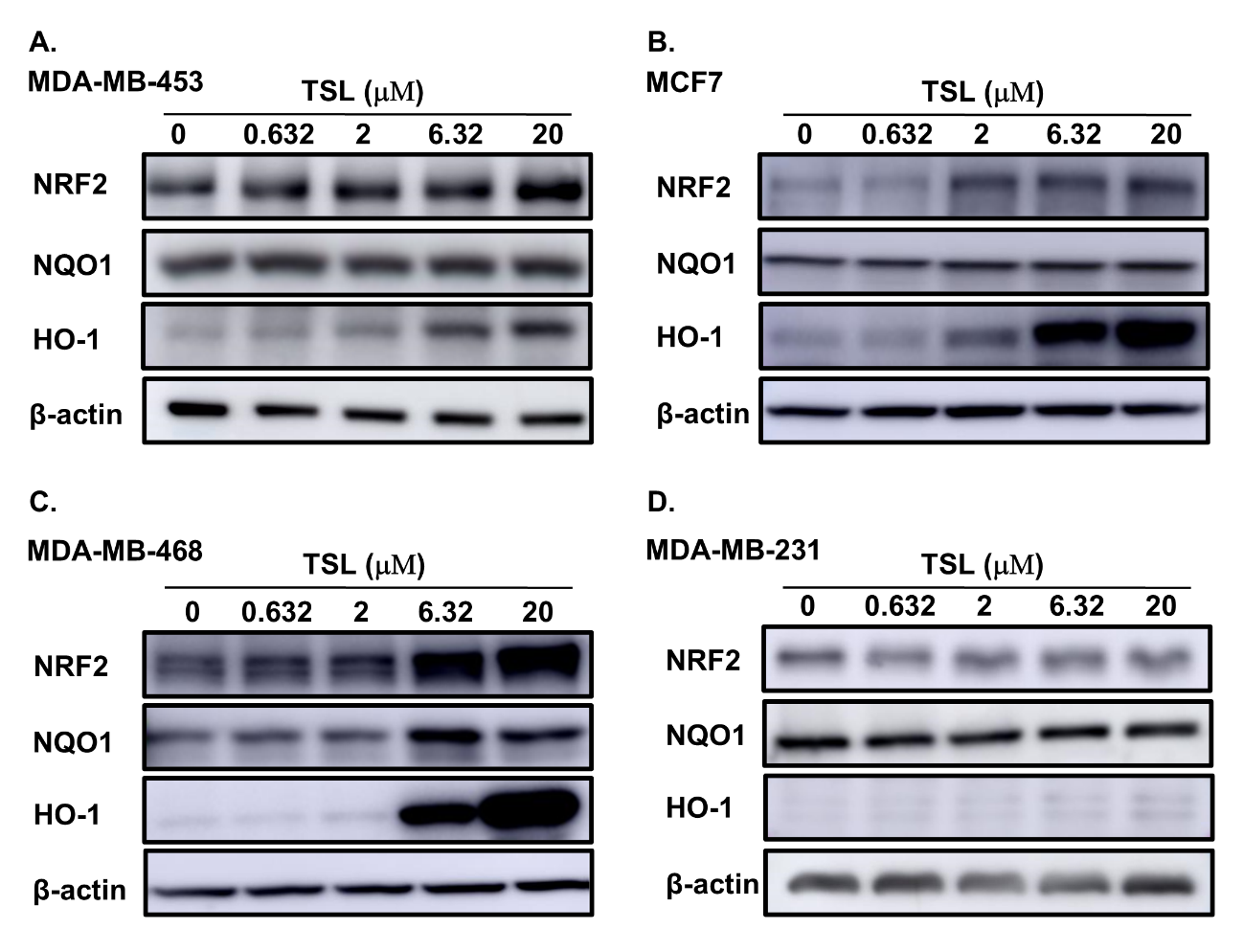


Supplementary Figure 7. Tanshinlactone activates NRF2 in other sensitive breast cancer cell lines MDA-MB-453, MCF7, and MDA-MB-468 compared to insensitive cells MDA-MB-231. Immunoblot analyses of NRF2 and its target proteins NQO1 and HO-1 in MDA-MB-453 (A), MCF7 (B), MDA-MB-468 (C), and MDA-MB-231 (D) cell lysates treated with TSL (0, 0.632, 2, 6.32, 20 μM) for 48 h. Representative immunoblots are from three independent experiments (n=3).

Supplementary Movie 1. Quick time movie showing tanshinlactone induces extreme cytoplasmic vacuolization in breast cancer cell lines ZR-75-1 and ruptures the cells following the accumulation of large cytoplasmic vacuoles.
